# LNA blockers for improved amplification selectivity

**DOI:** 10.1038/s41598-023-31871-7

**Published:** 2023-03-24

**Authors:** Jaime Prout, Michael Tian, Alicia Palladino, Jason Wright, John F. Thompson

**Affiliations:** Department of Genomics and Computational Biology, Homology Medicines, Inc., Bedford, MA 01730 USA

**Keywords:** Genetics, Molecular biology

## Abstract

LNA-containing oligonucleotides bind DNA more tightly than standard DNA, so they can interact with targeted sequences and affect multiple processes. When a desired DNA is present at low concentrations relative to nearly identical undesired DNAs, LNAs can block amplification of unwanted DNAs. Using a short rAAV and synthetic DNA sequence as a model, we studied the length, number, and positioning of LNA bases to improve blocker effectiveness. Oligonucleotides 18–24 bases long with LNAs at every other position were most effective. Highly degenerate targets were used to characterize the impact of mismatches on blocking. Mismatches at LNA ends had little impact on blocking activity. Single and double mismatches were tolerated with longer blockers, especially if the mismatches were near LNA ends. Shorter LNAs were more selective, with > 1 mismatch preventing effective blocking. Neither the strand to which a blocker bound nor the distance between the blocker and priming sites greatly impacted blocking efficiency. We used these findings to design blockers of wild-type DNA versus the single-base *A1AT* PiZ allele. Blockers are most specific when the mismatch is located away from the LNA 5′ end. Pairs of partially overlapping blockers on opposite strands with a centrally-located mismatch have maximal activity and specificity.

## Introduction

Locked Nucleic Acid (LNA) nucleotides are identical to natural nucleotides except for a methylene bridge spanning the deoxyribose sugar^1,2^ which makes them more stable in double-stranded structures and more resistant to degradation^3^. The higher melting temperatures (T_m_s) of oligonucleotides that include LNA bases provide greater specificity and new functions. They have been used successfully as PCR primers/probes^4–6^, as antisense reagents^7–9^, as selective binders for distinguishing single-nucleotide variants^10–18^, as agents for selective capture/degradation^19,20^, and as polymerization/splicing blockers^21–28^. Each of these roles requires that the LNA bind tighter than the corresponding pure DNA, but some functions may also require additional attributes that could be affected by the number and location of LNA bases within the oligonucleotide. Furthermore, proteins may interact with LNAs differently than with standard DNAs, dictating whether LNA or DNA should occupy a particular position.

While LNA blockers have been used in multiple situations, those studies have little commonality to provide insight into preferred designs. Some use chimeric^11–13,15,21,23,26^ or pure^10,17,27^ LNAs of 16 nt or shorter, while others use chimeric LNAs of 20 nt or longer^16,22,24^ For 20-mers, there are more than one million possible LNA-DNA configurations for each of the more than 1 trillion possible sequences. In addition, functional predictions of how different LNAs will perform as primers, blockers, or in other roles are even less well characterized because additional factors beyond the well-studied T_m_^29–33^ may play a critical role in how different LNAs perform^34–36^.

Blocking with LNAs is not the only method that has been used to prevent the amplification of majority DNAs when the detection of minority DNAs is desired. Other nucleic acid analogs like Peptide Nucleic Acids (PNA) selectively block amplification^37,38^. Choice of PNAs versus LNAs for a particular application will depend on the details of the system. LNAs have the advantages of lower cost and the ability to make longer molecules to reduce the number of perfect binding sites in complex genomes. PNAs can bind more strongly and are not susceptible to nucleases. A concern for PNAs can be specificity where the shorter PNAs may bind multiple genomic sites and potentially interfere with other DNAs of interest. In our case, we often wish to amplify DNAs of several kilobases or more arising from multiple genomic regions. Knowing that blockers will not interfere with DNAs elsewhere in the genome can be important. Thus, the more predictable and selective LNAs are preferred in our application though PNAs can be suitable in other situations.

One method for classifying blockers is whether they prevent the annealing of a PCR primer or prevent the elongation of that primer^39^. Annealing blockers are often used when the priming site for the desired amplicon is different by only one or by a small number of bases relative to the undesired amplicon. When few differences are present, shorter LNAs are often used to enhance the difference between perfect and imperfect binding. When the regions available for priming of the desired and unwanted DNAs are identical, annealing blockers cannot be used. Instead, sequences specific to the interior of the undesired amplicon must be bound by an elongation blocker. Our aim was to preferentially amplify integrated recombinant Adeno-Associated Virus (rAAV) while preventing the amplification of episomal copies of the transduced rAAV. Episomal DNA contains the same sequences as integrated DNA^40^, so elongation rather than annealing blockers must be used. Because other rAAVs and DNAs of interest may contain different sequences and hence need different amplification conditions, it is helpful to understand how to design effective LNA elongation blockers for different contexts and applications.

## Results

**Figure 1:**
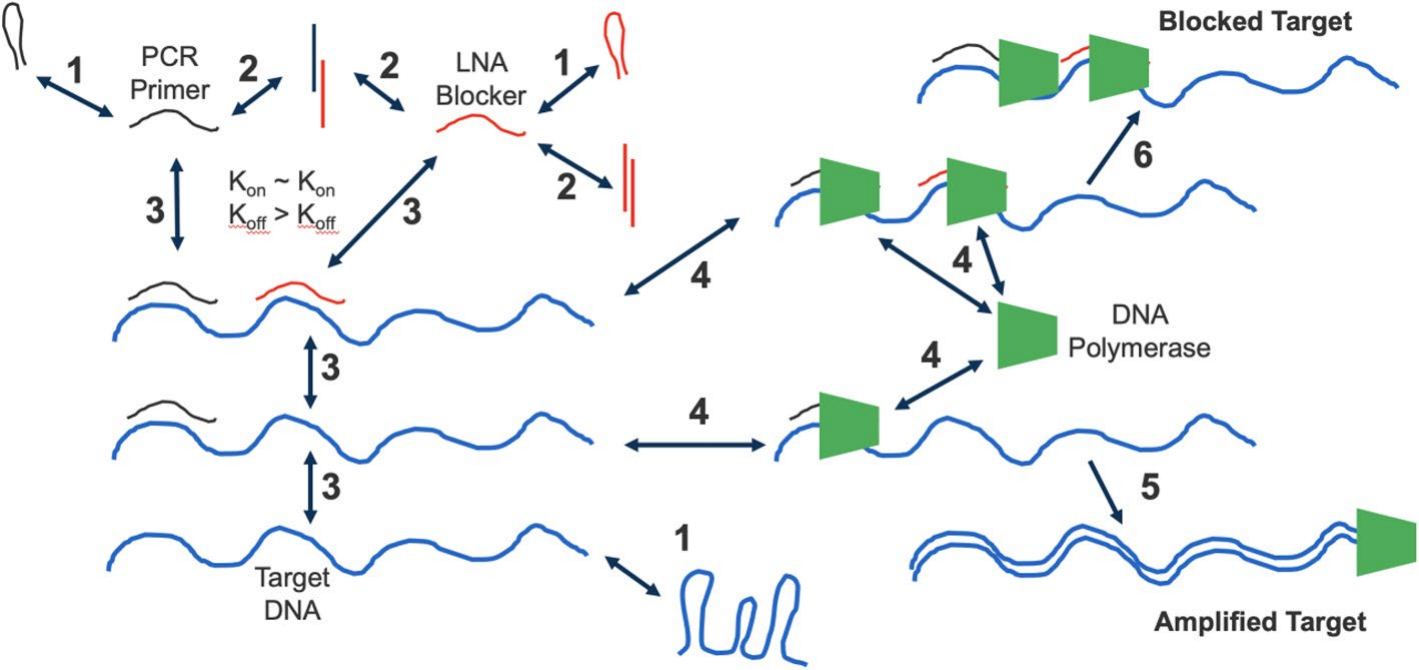
Potential interactions among intended reactants during PCR blockage. Potential intra- and inter-molecular interactions are shown for the PCR primer (black), the LNA blocker (red), the target DNA (blue), and the DNA polymerase (green). The DNAs/LNA can undergo folding into interfering secondary structures (1). The primer/blocker can potentially interact in a non-productive complex (2) with each other or in a productive or non-productive complex with the target DNA (3). LNAs have a slower off-rate versus DNAs^46^. The DNA polymerase can then bind the primer/blocker/target complex (4) and either extend the primer to generate an Amplified Target (5) or, if polymerization is halted by the LNA when the LNA is bound to the target DNA, a Blocked Target (6).

The process of blocking amplification involves many individual components. As diagrammed in Fig. 1, the priming and blocking oligonucleotides and the target DNA could all potentially participate in unimolecular, bimolecular, and higher-order interactions. During primer/blocker design, sequences are picked that minimize internal secondary structure and the formation of homo/heterodimers. The amplifying polymerase could bind to both oligo/target complexes and either stabilize or destabilize those interactions, with or without the additional complexity of polymerization. In addition to these potential interactions, there is also the likelihood that any component could interact with other DNAs in the mix because the reason for wanting to block the target DNA is to improve the signal relative to other related DNAs present in the complex mixture.

**Figure 2:**
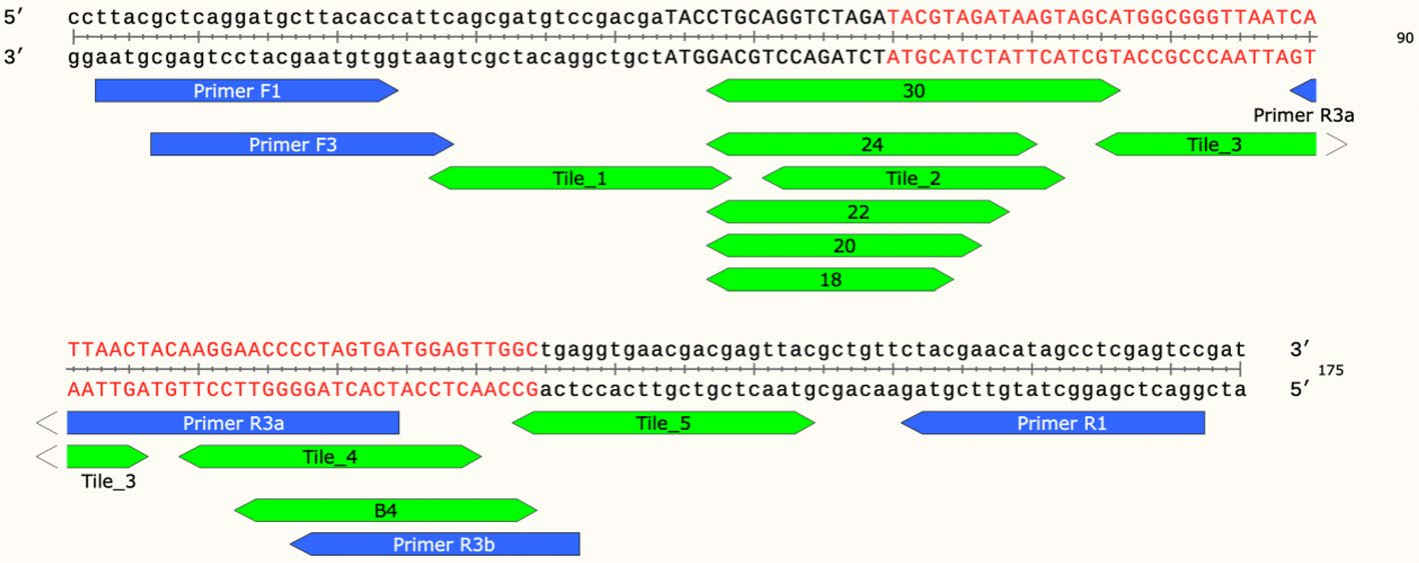
Template_0 target DNA and primer/LNA binding locations. The target oligonucleotide to be blocked (Template_0) includes a left primer region (1–43), a multiple cloning site (MCS) with SbfI, XbaI, and SnaBI sites (44–59), AAV2 sequence (4495–4558 in http://www.ncbi.nlm.nih.gov/nuccore/AF043303.1, 60–124, shown in red) and a right primer region (125–175). Two forward primer sequences on the left, F1 and F3, and three reverse primers on the right, R1, R3A, and R3B (blue arrows) were used to amplify the sequence with or without LNA blockers. Each LNA blocker (green double arrows) was made in four versions, two corresponding to the top strand (F, forward) and two corresponding to the bottom strand (R, reverse). The pair of F LNAs and the pair of R LNAs were synthesized starting with LNAs at either position 1 or position 2. These are listed individually in Table 2 and Table S1.

The large number of potential substrates and conditions prevent a complete analysis of all LNA lengths and sequences. For these experiments, we have restricted the total length of the LNA/DNA chimeras to those long enough to bind and provide reasonable specificity within the human genome (≥ 16 nt) while not so long as to allow binding to occur despite the presence of multiple mismatches. For the initial experiments (Table 1), the target DNA was a plasmid containing a sequence derived from a multiple cloning site and episomal rAAV^40^. Subsequent experiments (Table 2) used a synthetic 175mer DNA (Template_0) with the same targeted sequence flanked by different priming sequences (Fig. 2). Additional experiments used other DNA targets as described in Table S1. Generally, we used DNA polymerase GXL that lacks 5′ > 3′ exonuclease activity. All LNA-containing oligonucleotides were capped at both the 5′ and 3′ ends to minimize degradation and prevent extension as a primer. Initially, some LNAs were made with phosphorothioate linkages to prevent degradation. No effect was seen (data not shown), so later LNAs did not include such linkages.

**Table 1:**
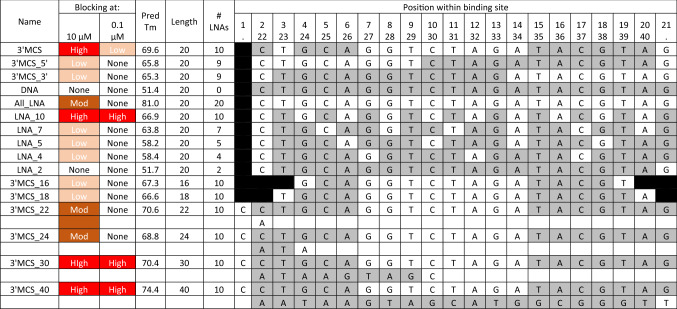
Qualitative assessment of LNA/DNA blocking efficiency.

Positions with LNAs are in white, positions with DNAs are in gray, and positions with no nucleotides are blacked out. Using a plasmid as the target DNA, different LNA/DNA chimera concentrations were added to the PCR mix at either 0.1 µM or 10 µM, and the product was analyzed via quantitative capillary electrophoresis. Blocking was assessed visually to determine relative efficiency. T_m_s were calculated using default conditions at https://www.idtdna.com/calc/analyzer.

**Table 2:** Blocking efficiency.

Name	Oligo start	Oligo end	Primers F1/R1		Primers F3/R1		Primers F1/R3A		Primers F1/R3B	
			Primer/blocker gap	% Blocking	Primer/blocker gap	% Blocking	Primer/blocker gap	% Blocking	Primer/blocker gap	% Blocking
Tile_1F1	27	48	2	98	− 2	99				
18_F1	47	64	22	73						
18_F2	47	64	22	89						
20_F1	47	66	22	68						
20_F2	47	66	22	83						
22_F1	47	68	22	58						
22_F2	47	68	22	95						
24_F1	47	70	22	91						
24_F2	47	70	22	97						
30_F1	47	76	22	90						
30_F2	47	76	22	94						
Tile_2F1	51	72	26	27	22	25				
Tile_3F1	75	96	50	82	46	83				
Tile_4F1	99	120	74	87	74	88				
B4_F1	103	124	78	98						
B4_F2	103	124	78	99						
Tile_5F1	123	144	98	90	94	91				

Name	Oligo start	Oligo end	Primers F1/R1		Primers F3/R1		Primers F1/R3A		Primers F1/R3B	
			Primer/blocker gap	% Blocking	Primer/blocker gap	% Blocking	Primer/blocker gap	% Blocking	Primer/blocker gap	% Blocking
Tile_5R1	144	123	6	96	6	96				
B4_R1	124	103	26	35						
B4_R2	124	103	26	49						
Tile_4R1	120	99	30	15	30	16	− 31	10	− 13	16
Tile_3R1	96	75	54	68	54	88	− 7	96	11	87
30_R1	76	47	74	59			13	99	31	99
30_R2	76	47	74	68						
Tile_2R1	72	51	78	46	78	50	17	87	35	72
24_R1	70	47	80	93			19	96	37	92
24_R2	70	47	80	98						
22_R1	68	47	82	80			21	94	39	91
22_R2	68	47	82	95						
20_R1	66	47	84	78			23	92	41	89
20_R2	66	47	84	86						
18_R1	64	47	86	94			25	81	43	62
18_R2	64	47	86	74						
Tile_1R1	48	27	102	94	102	91	41	98	59	95

Positions of LNA blockers versus Template_0 are listed. More detailed information on each blocker can be found in Table S1. Four sets of primers (shown in Fig. 2) were used with Template_0 and the indicated blockers added at 1 µM. The length of the gap between the elongating primer and the blocker is shown, as well as the efficiency of blocking for each primer set. Blockers identical to the top strand (F) are in the top half of the table, and blockers identical to the bottom strand (R) are in the bottom half of the table.

Qualitative assays were run at varying LNA concentrations to identify where to focus with length and the number/positioning of LNAs (Table 1). For these studies, varying numbers of LNAs were situated in the middle, at either end, or throughout the oligonucleotide. The LNAs were added to PCR reactions at 0.1 µM or 10 µM, and all were targeted to the same DNA site for consistent comparisons. We found that maximum effectiveness occurred when chimeric molecules were ≥ 20 nt and half of the positions were substituted with LNAs. Furthermore, an even distribution of LNAs throughout the molecule (LNA_10) was more effective than LNAs clustered together (3′MCS, 3′MCS_5′, and 3′MCS_3′), so all later LNAs contained alternating LNA/DNA.

Based on the results in Table 1, a new set of 18–30 nt blockers was synthesized with alternating DNA/LNA nucleotides (Fig. 2, Table 2, and Table S1). LNAs were made with sequences identical to the top (forward, F) strand and the bottom (reverse, R) strand of the 175 nt synthetic Template_0 (Fig. 2). In most cases, pairs of oligos were made, varying with respect to whether the first LNA was placed at position 1 or position 2. Oligonucleotides with names ending in 1 started with the first nucleotide as LNA, while oligonucleotides with names ending in 2 started with the second nucleotide as LNA with DNA/LNA alternating thereafter. All chimeras with LNAs starting with the second position had higher predicted T_m_s than the equivalent molecules starting with LNA in the first position (Table S1). Individual LNAs were used to block amplification at 1 µM, with their effectiveness measured using quantitative capillary electrophoresis relative to samples with no LNA added (Fig. S1, Table 2). Effectiveness varied significantly among oligonucleotides. In most cases (10 of 12 tested), blockers with LNAs starting in the second/even positions were more effective than the same blockers with LNAs in the first/odd positions. Nearly half (15 of 34) of the blockers tested with primers F1/R1 yielded > 90% blocking, and another 6 blocked > 80%.

The initial results with the F1/R1 primer pair suggested that blockers separated on the same strand from the primers by 20–40 nt did not work as well as those positioned further away. The length of DNA gaps between the primers and blockers was changed to determine whether this affected blocking efficiency. Primer R1 was replaced with primers R3A and R3B that were 61 and 43 nt closer to the blocker binding sites to generate altered primer/blocker spacing. Blocker effectiveness did not change when the new primers were 20–40 nt away from the blocker, suggesting that, if there is a gap distance effect, it is not strong. Similarly, moving the primer by 4 bp to change helical orientation on the forward strand (primer F1 versus F3) had minimal effect on blocking (Table 2).

While the blocking efficiency of LNAs is a critical feature, some applications require specificity so that non-targeted sequences are minimally affected. To assess how target mismatches affect blocking efficiency, Template_D was synthesized with a blocker binding region containing multiple partially degenerate positions located at the site bound by blockers 18_F2, 18_R2, 20_F2, and 20_R2 (Fig. S2). Template_D had a different sequence relative to Template_0 beyond the 20 nt identical region bound by these blockers (discussed in Fig. S2 legend). The degeneracy introduced by using a mixture of nucleotides during synthesis allows the creation of a highly complex pool of DNA molecules with a wide range of variants that can be used as a substrate for determining the effect of DNA mismatches on LNA blocking. This pool contained potentially over 1 trillion different DNA molecules with randomly situated variants within the degenerate region. Because the synthetic nucleotide pool favored the reference sequence (79%), molecules averaged only 4 variants each. In addition to the 18- and 20-mers that could be tested on both Template_0 and Template_D, we also wanted to test Template_D with a longer blocker. A new pair of 24 nt LNAs was made (24D_F2 and 24D_R2). Even though the 24D blockers were 24 nt long, they covered only 21 degenerate positions because three positions bound by 24D consist of 100% reference sequence.

When there is a constant level of degeneracy in DNA, the frequency of variants within a given length can be predicted mathematically using classic combination/permutation equations (https://en.wikipedia.org/wiki/Combination). When R is the proportion of correct reference sequence and L is the length of the degeneracy, the frequency of a perfect reference sequence (no variation) is ${R}^{L}$. For DNA synthesized to be 79% Reference/21% Mismatch, the percent of reads matching the reference sequence perfectly is predicted to be 1.4% for an 18mer and falls to 0.7% for a 21mer. For predicting the number of variant sites in all molecules, the expected frequency for any given number of mismatches (MM) in the target region of length L can be calculated from the equation:

$$\% \text{ reads with mismatches (MM)} =100\times {R}^{L-MM}\times {(1-R)}^{MM}\times \frac{L!}{MM!\times (L-MM)!}$$

**Figure 3:**
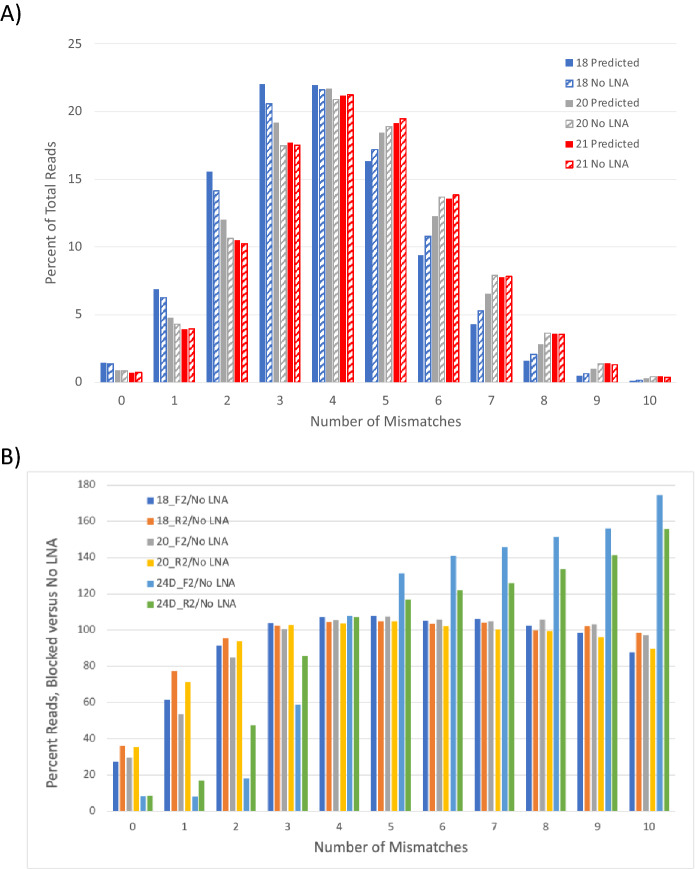
Predicted and observed mismatch read frequencies. (**A**) The predicted mismatch frequency (solid fill) and observed variant read frequency for unblocked samples (hatched fill) as a percent of total reads normalized by sample for 0–10 variants is shown for degenerate DNA lengths of 18 (blue), 20 (gray), and 21 (red) nt. These predictions were made using the combination equation in the text where R = 0.79, L = 18 or 20 or 21 and MM = 0–10. > 99% of all molecules with these degeneracy lengths and 79% reference sequence should have 10 or fewer variants so higher variant values are not shown. Even though the 24D blockers are 24 nt long, a length of 21 is used for its calculations because only 21 of the 24 positions on the target DNA are degenerate. The calculated frequencies are nearly identical to the observed variant frequencies in DNA reads when the amplification is not blocked by LNAs (Table S2). (**B**) For forward (F2) or reverse (R2) blockers covering 18, 20, or 21 degenerate positions, the percent of reads for each sample versus no LNA present is shown for each mismatch value. The number of mismatches allowed while still retaining blocking activity varies by length.

For example, to determine the frequency of molecules with 3 mismatches in a 20mer that was 79% reference sequence, one would calculate 100 * (0.79)^17^ * (0.21)^3^ * (20!)/((3!) * (17!)) yielding 19.2%, meaning that 19.2% of all 20mers should have exactly three mismatches somewhere within their sequence. The predicted read frequencies as a function of the number of mismatches for 18, 20, and 21 degeneracies are shown in Fig. 3A. The frequencies for the 24D LNAs were calculated with 21 degeneracies because three template positions bound by the LNAs were reference, not degenerate. To compare the predicted frequency with the actual frequency, the target DNA pool was amplified with primers that bound the constant primer region outside of the degenerate regions. After barcoding, the amplified samples were sequenced using a MiSeq and the frequency of variant positions compared to the predicted results. The mismatch frequency for the samples with no LNA blocking closely mirrors the calculated values for 79% reference/21% mismatch (Fig. 3A and Table S2). Values were calculated only up to 10 mismatches because, with these synthetic parameters, more than 99% of DNA molecules have 10 variants or fewer.

To determine the impact of mismatches on blocking, amplifications of the complex pool of DNA sequences that make up Template_D were performed with either no LNA or individual LNAs at 1 µM. The primers used to amplify Template_D each bound identical, non-degenerate regions of the DNAs while the blockers had trillions of different sequences to which they could bind. The ability of LNAs to bind certain sequences caused blocking of those DNAs while others amplified normally. If variants in the target pool are differentially affected by LNA blockers, the read distribution should change accordingly. When blocked samples were examined (Fig. 3B), all 18 and 20 nt blockers had 27–36% as many reads with no mismatches as the unblocked samples, while the 24 nt blockers had only 8.4–8.6% as many reads with no mismatches as unblocked samples, showing that specific blocking had occurred, and the amount of blocking was length dependent. The read frequency for each mismatch was very similar when 18_F2/18_R2 and 20_F2/20_R2 were compared, indicating that the strand being blocked does not matter with this target. There is a greater deviation between 24D_F2 and 24D_R2, but this may be due to the asymmetric location of degenerate positions in the two blockers. 24D_R2 has three fixed target positions near its 5′ end, while the fixed positions for 24D_F2 are near its 3′ end.

For the 18 and 20 nt blockers, there were 54–77% as many reads with 1 mismatch relative to no LNA. There is only a slight difference in reads (85–96% as many reads relative to no LNA) with 2 mismatches relative to no LNA, indicating that any mismatches lead to poor blocking at these LNA lengths. All blocking activity was lost with three or more mismatches for 18 and 20 nt blockers. With the 24 nt blockers, there was significant blocking with 0, 1, and 2 mismatches. There was lower but measurable blocking (59–86% as many reads relative to no LNA) with 3 mismatches. This indicates that the specificity of blocking is dependent on the length of the blocker and longer LNAs are capable of blocking to some degree, even in the presence of up to three suitably placed mismatches.

**Figure 4:**
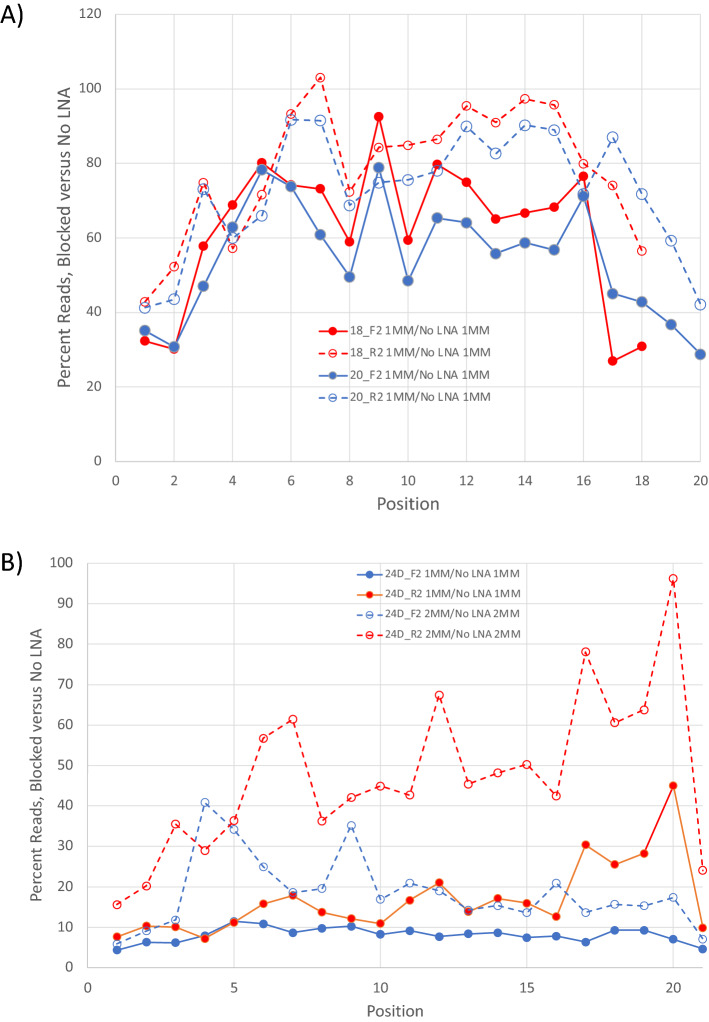
Blocking as function of position. (**A**) The relative read frequency for amplification with blockers versus amplification with no LNAs is shown as a function of position. In panel A, the frequency of reads with single mismatches is shown for 18 (red) and 20 (blue) nt LNA blockers. Blockers identical to the forward strand are shown with solid lines/symbols, and blockers identical to the reverse strand are shown with dashed lines/open symbols. The overall shape for all forward primers and for all reverse primers is similar. (**B**) LNAs blockers that cover 21 degenerate positions are shown with data for reads for one mismatch (1MM, solid lines/symbols) and for reads with two mismatches (2MM, dashed lines/open symbols). Blockers identical to the forward strand are shown in blue, and the reverse strand in red. Blocking in the presence of mismatches is not necessarily desirable as it indicates a lack of selectivity.

To assess the relative importance of different positions within the LNAs for blocking specificity, the locations of mismatches within all reads with a single mismatch were examined. The positional frequency of single mismatches in the No LNA sample relative to all reads in the sample is uniform as each mismatch has no effect on amplification (Table S2). The frequency of single mismatch reads by position relative to all reads in 18 and 20 nt blocked samples varies significantly and is shown in Fig. 4A. The frequencies are given as a percentage relative to the values observed in the unblocked sample. Because effective blocking was observed with both one and two mismatches with 24D_F2 and 24D_R2, the positional effects for both one and two mismatches are shown for those blockers (Fig. 4B). If all positions are equally important for blocking, all blocked samples would have the same percentage across the length of the LNA. Indeed, that is the observation with the 24D_F2 LNA with a single mismatch: it does not matter where the mismatch occurs, there is 90–95% blocking, independent of position. For all other conditions with one or two mismatches, there is a positional dependence on blocker effectiveness. Positions very close to either end of the blocked region have lower relative read frequencies than the more centrally located positions. The lower read frequency for mismatches near the blocker ends means templates with those mismatches are blocked despite the mismatch, so they are less represented among all reads. If selectivity of blocking is desired, significant blocking in the presence of mismatches is not a good thing. Though the position of the mismatch matters, the identity of the base substitution at a given position is generally irrelevant. At most positions, all mismatches have similar blocking effects (Table S3). There are some differences but no obvious trends.

An additional common feature among blockers that did not perform well was the presence of an A on the same strand adjacent to the 5′ end of the blocker binding site. When the base was an A, the average blocking effectiveness was 42% (n = 5), while it was 84% for C (n = 16), 93% for G (n = 4), and 81% for T (n = 9). To test the same blockers with a different adjacent sequence, two new templates were made (Template_A1 and Template_A2, Figs. S3 and S4). Many LNA blockers shown in Table 2 had overlapping binding sites, so making a base change adjacent to one binding site created a mismatch within other binding sites. Only the subset of the original blockers that maintained perfect binding sites were tested on the new templates. For the blockers tested, all preceding non-As were changed to A, and all preceding As were changed to another base (Table 3, Figs. S3 and S4). The eleven changes from C/G/T to A resulted in an average decrease in efficiency of 21% while the three changes from A to G/T resulted in an average increase in efficiency of 15%. In addition to Template_A1 and Template_A2 which have uniformly altered sequences, it is also possible to use Template_D to address the question of adjacent sequence. Two of the blockers used, 18_R2 and 24D_R2, have a degenerate position adjacent to the 5′ end of their binding site on that target DNA. Thus, the frequency of each base prior to their binding sites can be examined for differences. There do not appear to be significant base-specific effects at those sites with Template_D; so, if adjacent bases have an impact, the effect is small, may be more complex than just a single base, or may be evident only in certain conditions.

**Table 3:** Impact of preceding base.

Name	Template 0	Sequence change	Template A1	Difference A1	Template A2	Difference A2
A > N						
30_R1	0.47	77 A > T	0.92	0.45		
Tile_4R1	0.31	121 A > G			0.16	− 0.15
B4_R1	0.21	125 A > G	0.37	0.16		
N > A						
18_F1	0.73	46 C > A	0.82	0.09		
Tile_1R1	0.94	49 C > A			0.60	− 0.33
Tile_2F1	0.27	50 C > A			0.27	0.00
Tile_4F1	0.87	98 C > A			0.37	− 0.50
Tile_5R1	0.96	145 C > A			0.85	− 0.11
Tile_3F1	0.82	74 G > A			0.50	− 0.32
B4_F1	0.98	102 G > A	0.61	− 0.37		
Tile_5F1	0.90	122 G > A			0.44	− 0.47
Tile_1F1	0.98	26 T > A			0.80	− 0.18
Tile_2R1	0.46	73 T > A			0.50	0.03
Tile_3R1	0.68	97 T > A			0.52	− 0.16

Fourteen blockers were tested on two templates, the original Template_0 and either Template_A1 or Template_A2. Both Template_A1 (Fig. S3) and Template_A2 (Fig. S4) have altered sequence adjacent to selected blocker binding sites. Below, the first three blockers involved a change from A to either T or G, while the last eleven blockers involved a change from C, G, or T to A. The initial blocking efficiency with Template_0 is shown in the second column and the second blocking efficiency is shown in the 4th or 6th column with all LNAs added at 1 µM.

A key component of the overall amplification and blocking process is the DNA polymerase. The experiments described thus far were carried out with GXL polymerase, which has 3′ > 5′ exonuclease activity, but no 5′ > 3′ exonuclease activity as some other polymerases have. Two additional polymerases were tested in parallel against a subset of LNA blockers carried out in conditions and temperatures recommended by the suppliers. As shown in Table 4, the two polymerases which lack 5′ > 3′ exonuclease activity, GXL and Q5, had similar blocking profiles. The polymerase that does exhibit 5′ > 3′ exonuclease activity, GoTaq, behaves differently. Template_U has no LNA binding sites, but it is blocked to the same extent as Template_O with 6 of the 8 blockers tested. There may be a small amount of specific blocking with the two best blockers, 30_R1 and 30_R2. Literature results for blocking of Taq have produced discrepant results^11,41^. Those experiments were run at different temperatures, so we also tested GoTaq with an extension temperature of 60 °C where LNA blocking was reported. We find specific blocking at that temperature though not as much as found with the other polymerases.

**Table 4:** LNA blocking as a function of DNA polymerase.

Polymerase >	GXL—68C		Q5—72C		GoTaq—68C		GoTaq—60C	
Template >	T_O (%)	T_U (%)	T_O (%)	T_U (%)	T_O (%)	T_U (%)	T_O (%)	T_U (%)
20_F1	72	35	64	23	26	37	37	0
20_F2	73	22	66	26	43	43	47	− 4
20_R1	69	24	32	27	40	39	47	− 3
20_R2	92	23	84	28	40	32	51	− 10
30_F1	92	40	89	29	44	40	38	− 6
30_F2	95	53	90	24	41	40	34	2
30_R1	98	41	97	22	53	42	37	− 14
30_R2	95	38	99	25	48	37	44	− 9

Eight blockers with moderate to high blocking effectiveness were evaluated with three different polymerases using the indicated extension temperatures. The manufacturers’ suggested buffer and cycling conditions were used for each polymerase except for when GoTaq was used with a 60 °C extension. Blocking was attempted with both Template_0 that contains a specific binding site and Template_U which did not contain any site recognizable by these blockers. Both templates were amplified using primers F1 and R1 with LNA added to 1 µM.

**Figure 5:**
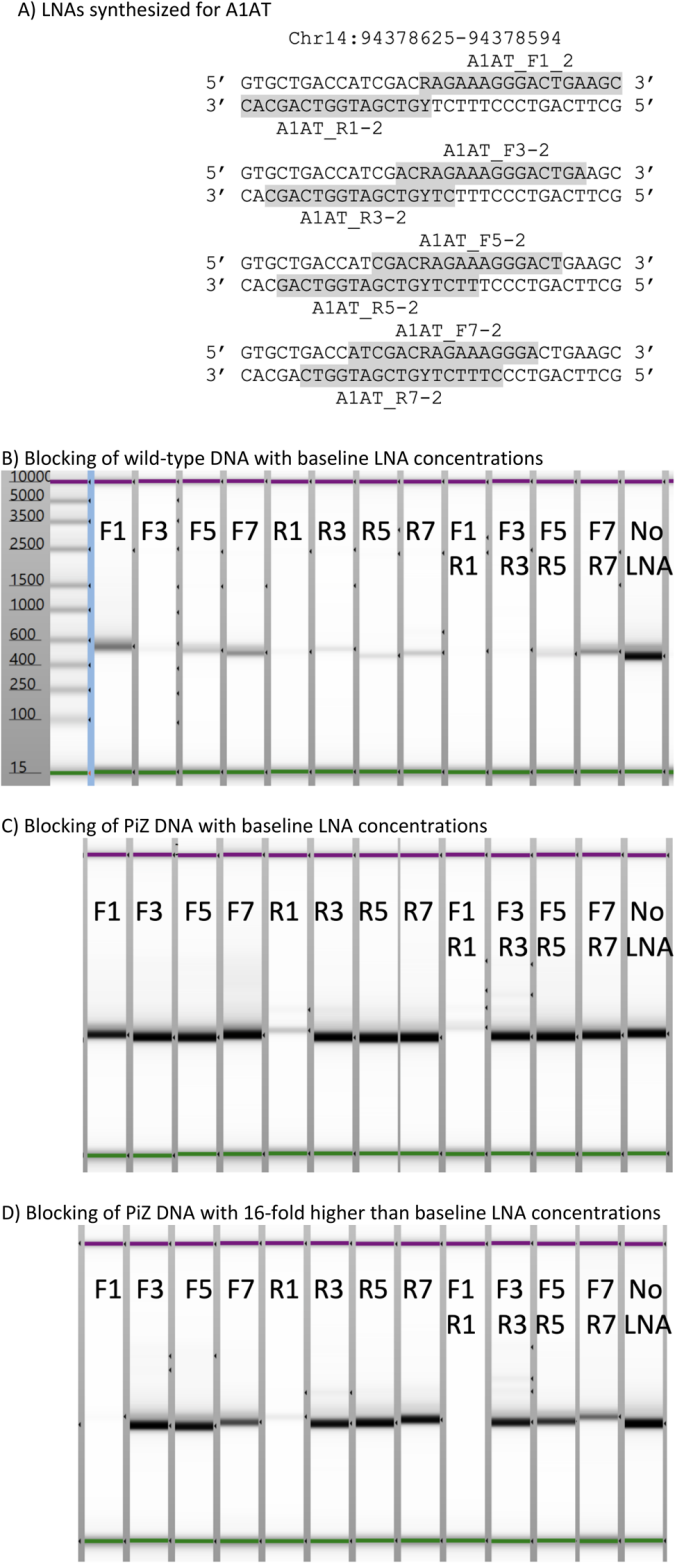
Blocking *A1AT*. (**A**) The sequence surrounding the PiZ allele in *A1AT* is shown (central base, R/Y). G/C is the wild-type sequence and A/T is the PiZ allele. LNAs are named with an F if identical to the top strand and R if identical to the bottom strand. The number adjacent to F/R indicates where the PiZ allele is in the LNA. Individual LNAs and how they overlap are shaded. Because each LNA varies by two in length, the alternating LNAs are positioned identically in these series. Based on potency in initial studies at 1 and 10 µM, concentrations were titrated to obtain good but not complete blocking at the lowest concentrations used. The lowest concentration for A1AT_R1-2 was 0.1 µM, followed by A1AT_F3-2, A1AT_R3-2, and A1AT_R5-2 at 0.2 µM, A1AT_F5-2 and A1AT_R7-2 at 0.5 µM, A1AT_F7-2 at 1 µM, and A1AT_F1-2 at 3.3 µM. (**B**) Blocking of wild-type DNA with LNA concentrations listed in (**A**). (**C**) Blocking of PiZ DNA with LNA concentrations listed in (**A**). (**D**) Blocking of PiZ DNA with LNA concentrations 16-fold higher than listed in (**A**). Data is not shown for the 16 × wild-type DNA because all LNAs completely blocked amplification.

To see if these design properties could be extended to another target of biological interest, LNA chimeras were designed to the wild-type allele of *A1AT* to see whether its amplification could be blocked specifically relative to the single base PiZ mutation which is linked to COPD and other respiratory diseases^42^. Four pairs of LNAs matching the wild-type sequence were made (Fig. 5A). These LNA pairs placed the base corresponding to the PiZ mutation at the 5′ end and at the third, fifth, or seventh position in from the 5′ end. This was done for both DNA strands (Forward and Reverse). All LNAs started at the second position and alternated through the molecule. Five of the eight were 16 nt long but the three with the lowest T_m_s were extended one additional base to make the T_m_s more similar (Table S1). The region around the PiZ allele was amplified from human genomic DNA using primers listed in Table S1 to generate a 646 bp product. LNAs were titrated versus wild-type DNA to find concentrations of each that would yield extensive but not complete blocking. These concentrations varied significantly and were independent of T_m_. The best *A1AT* LNA blocker, A1AT_R1_2, was 30 times more potent than the worst blocker, A1AT_F1_2. Based on this, genomic DNA with either wild-type or PiZ alleles was amplified with the eight individual LNAs as well as with each pair of corresponding F/R LNAs. As shown in Fig. 5, concentrations that yielded significant blocking of wild-type *A1AT* had no effect on PiZ *A1AT* except for A1AT_F1-2 and A1AT_R1_2 which blocked the PiZ DNA nearly as well as wild-type despite the mismatch at the 5′ end for both. The combination pairs all blocked wild-type DNA better than the individual blockers. When LNA concentrations were increased 16-fold, five LNAs blocked PiZ DNA amplification less than 30% (A1AT_F3-2, A1AT_F5-2, A1AT_R3_2, A1AT_R5_2, and A1AT_R7_2) while A1AT_F1-2 and A1AT_R1_2 blocked it more than 95%. More effective blocking of PiZ DNA was also observed with all combinations except for A1AT_F7_2/ A1AT_R7_2 which was similar to the individual blockers. This blocker pair overlaps each other by 13 nt so it is likely they will bind each other in solution, potentially affecting their ability to bind to both wild-type and PiZ DNA.

## Discussion

PCR has been used to routinely amplify rare DNA sequences from complex mixtures. When the desired DNA has unique sequence characteristics, high degrees of amplification are readily achieved. When the targeted sequence is overwhelmed by an excess of highly similar sequences, selective amplification may not be as straightforward, and detecting specific rare DNAs in a mix of nearly identical molecules can be challenging. For example, when seeking to identify rAAV DNAs that have integrated into the host cell genome, there can be a high level of episomal DNA, up to thousands of copies per cell, obscuring less frequent genomic integration events. Sherman et al.^24^ used 27–32 nt blockers with 9 LNAs to address a similar issue with retroviral and lentiviral genomic integrations. These viral integration studies encountered a less severe problem due to the absence of high copy episomal DNA. Similar scenarios, such as complex metagenomic samples, somatic mutations, multiple paralogs, repetitive sequences, or pseudogenes, can also be complicated by DNA ratios for which the desired amplification is challenging. In the rAAV scenario, unique sequences are present in the non-integrated segments of the episomal virus relative to the integrated version, providing wide latitude in sequence choice. In other situations, there may be only one or a small number of changes, limiting the flexibility in blocker choice.

Based on thermodynamic studies^29–33^, all the oligonucleotides with LNA substitutions we studied should stably bind the target DNAs of interest. While LNA binding is a minimal requirement, the positioning and number of LNAs may or may not affect function, so relying on binding alone to predict function would be ill-advised. A 50% fractional LNA content performed best in initial experiments, especially when LNAs were located throughout the blocker rather than clustered in one region. Our initial observations suggesting that alternating LNA substitutions are functionally superior is consistent with other studies that indicate special LNA binding properties with such an arrangement^43,44^. Blocking activity was generally better when the LNAs started at the second position rather than the first.

To allow analysis of trillions of different targets, a highly degenerate pool of DNAs was synthesized for blocking studies. For DNA with 18–21 degenerate positions with the potential for all four bases at each position, this was equivalent to testing up to 4 trillion different targets simultaneously. Because the target DNA was synthesized to favor the reference sequence, molecules averaged four variants each, but could have anywhere from 0 to 21 variant positions. It is unlikely that most molecules with > 10 variants are represented because the pool was designed to test DNAs with fewer variants. We found that the exact sequence of mismatches did not generally have an effect at any blocked position. However, the position of the mismatch on the template does matter. Reads with mismatches very close to either end of the blocker were found far less often than more centrally located mismatches, indicating that mismatches at the ends still allowed less specific blocking, while more interior mismatches prevented blocking. If blocking of both perfect and slightly imperfect matches is not an issue, this positional effect will not matter.

However, when the intent is to distinguish between two DNA sequences that differ by only a single base, the positional effect is critical. Placing the mismatch away from the blocker ends is necessary if there is to be good blocking with a perfect match and no blocking with a single mismatch. We used the PiZ single base mutation in the *A1AT* gene as an example for evaluating the generalizability of these findings. Four pairs of LNAs that placed the mismatch position at the 5′ end or 3, 5, or 7 nt into the LNA confirmed that internal LNA positions are superior to the 5′ end with respect to specificity. While all LNAs block the wild-type allele, the 5′ end mismatch also blocks PiZ allele DNA with nearly the same effectiveness. When LNAs binding to both strands are used, improved blocking is observed. Of the LNAs we tested, the combination of A1AT_F7-2 and A1AT_R7-2 had the greatest overlap between them, 13 nt. Even with this high degree of overlap that could lead to stable binding to each other, blocking of the targeted allele remained strong though with slightly reduced specificity toward the mutation. The most effective and specific blockers had the mismatch located 3 or 5 nt from the 5′ end.

Previously, there have been reports that polymerases with 5′ > 3′ exonuclease activity eliminate LNA blocking activity^11^, while others have found no effect^41^. In our hands, Taq polymerase with 5′ > 3′ exonuclease activity is blocked non-specifically by LNAs when amplification is carried out at the recommended extension temperature. Amplification is inhibited 26–53% (Table 4) when there are specific target binding sites present (Template_0) and 32–48% when there are no LNA binding sites present (Template_U). There may be some specific blocking with the best blockers, 30_R1 and 30R_2. GXL and Q5 polymerases, which both lack 5′ > 3′ exonuclease activity, are non-specifically inhibited to a lesser degree and have much higher specific inhibition when there is a target binding site. If the amplification conditions that produced disparate results in the literature are compared, the most notable difference is the extension temperature during amplification. When primers are extended at 72 °C^11^, polymerases with 5′ > 3′ exo activity exhibit no blocking while extension at 60 °C^41^ retains blocking. We can mimic both results by adjusting the Taq extension temperature. Previous examination of Taq and the Stoffel analog, which lacks 5′ > 3′ exo activity, showed marked differences in temperature dependence of single versus double-stranded template replication^45^ that may explain why 5′ > 3′ exo activity matters in some situations but not others. Other polymerase-specific properties like fidelity and processivity were not tested but could also be envisioned to affect blocking. Thus, if certain polymerases are required for a specific application, it is necessary to test them to ensure that they can be effectively blocked by the desired LNAs.

The factors identified here are not guarantees of LNA blocker success, but they provide a guide when designing amplification blockers. The widely different concentrations of the *A1AT* LNAs required to achieve similar blocking on overlapping sequences highlight the remaining design unpredictability. Blockers of 18–24 nt with alternating LNAs beginning in the second position are a reasonable starting point when selectivity is not critical. If high selectivity for single base changes is needed, shorter oligos should be used (16–18 nt), while longer ones (24 nt) can be used if one or two mismatches are acceptable. For more complete blocking, both DNA strands should be targeted so that neither undesired strand would be synthesized. If only one or a few sequence differences can be exploited and selectivity is needed, it would be best to place mismatches away from the LNA ends.

## Materials and methods

### PCR conditions and blocking efficiency

PCR reactions were carried out with GXL polymerase (Takara), Q5 polymerase (NEB), or GoTaq polymerase (Promega) with the addition of specified LNAs to the listed final concentrations. The target DNA used for Table 1 results was 0.001 ng plasmid DNA^40^. The target DNA used for *A1AT* in Fig. 5 was Promega human genomic DNA for the wild-type allele or DNA from a human cell line containing the PiZ allele. The target DNA used for all other experiments was synthetic DNA templates (0.001 ng) whose sequences are listed in Table S1. Most experiments were carried out with the DNA polymerase GXL and cycling parameters of 18 cycles of 98 °C for 10 s, 55 °C for 15 s, and 68 °C for 30 s. In Table 4, other polymerases were also used with cycling parameters for Q5 of initial denaturation of 98 °C for 30 s followed by 18 cycles of 98 °C for 10 s, 55 °C for 15 s, and 72 °C for 30 s and a final extension at 72 °C for 2 min. Cycling parameters for GoTaq consist of initial denaturation of 95 °C for 2 min followed by 18 cycles of 95 °C for 30 s, 55 °C for 15 s, and 60 °C or 68 °C for 30 s and a final extension at 60 °C or 68 °C for 5 min. All experiments included controls with no LNA added. Primers, LNAs, and non-degenerate templates were ordered from Integrated DNA Technologies (IDT), and the degenerate template (Template_D) was ordered from Genewiz/Azenta. All template, LNA, and primer sequences are provided in Table S1. The Template_0 sequence was derived from a plasmid which was used as the template DNA for the reactions in Table 1. That sequence included a synthetic multicloning region and a region of AAV DNA. PCRs with LNAs, primers, and templates were tested as described in Tables 1 and 2. For the Table 2 experiments, at least 3 replicates were performed for each LNA/Template combination. From each PCR reaction, 1 µL was run using quantitative capillary electrophoresis according to the recommended protocol for D1000 tapes (Agilent) (Fig. S1).

### Evaluating primer-LNA binding position

To determine if the distance from the primer to the LNA binding site was important, a forward primer, F3, was designed for use in conjunction with primer R1 and tested with tiling LNAs 1–5 directed to either strand with Template_0. Additionally, two reverse primers, R3a and R3b, were designed and tested in conjunction with primer F1 and tested with LNAs 18_R1, 20_R1, 22_R1, 24_R1, 30_R1, and tiling LNAs 1-4R with Template_0.

### Evaluating the base prior to LNA binding region

To investigate whether the base just prior to the LNA binding region was important, two DNAs, Template_A1 (Fig. S3) and Template_A2 (Fig. S4), were synthesized with a series of single base changes relative to Template_0. With primers F1 and R1 and using conditions stated above, LNAs 18_F1, 30_R1, B4_F1, and B4_R1 were evaluated with Template_A1 and LNAs Tile_1F, Tile_1R, Tile_2F, Tile_3F, Tile_3R, Tile_4F, Tile_4R, Tile_5F, and Tile_5R were evaluated with Template_A2.

### Quantitative capillary electrophoresis data analysis

For experiments where Agilent quantitative capillary electrophoresis was used to measure the signal, the concentration was recorded for the band of interest for each sample. Replicates were averaged, and percent blocking was calculated with the formula

$$\%\, Blocking= \left(1- \frac{[average\, LNA\, signal]}{[average\, no\, LNA\, signal]}\right) \times 100$$

### Degenerate template sequencing

LNAs 18_F1, 18_F2, 18_R1, 18_R2, 20_F1, 20_F2, 20_R1, and 20_R2, 24D_F2, 24D_R2, and No LNA were evaluated using Template_D where the LNA binding region was synthesized with a degenerate sequence with 79% reference base/7% each non-reference base to determine the role of mismatches in the target sequence. PCR was performed as above, except primers 66Set1 and R1-2 were used. These primers included sequences compatible with Illumina sequencing. Replicates were pooled and purified using Monarch PCR and DNA Cleanup Kit (NEB). A barcoding PCR was performed with 1 µL of each purified PCR1 using GXL polymerase with the following conditions, 98 °C for 2 min, 25 cycles of 98 °C for 10 s, 55 °C for 15 s, 68 °C for 30 s, and a final extension at 68 °C for 3 min. Barcoded products were purified again, and 1 µL run via Agilent Quantitative Capillary Electrophoresis according to the recommended protocol for D1000 tapes to check for clean products and calculate molarity. Products were then pooled equimolarly, and 50% PhiX was added for library complexity. The DNA pool was melted, diluted, and loaded onto an Illumina MiSeq following the standard V2 300 Cycle kit protocol.

### Sequencing data analysis

Demultiplexed Fastq files were collected from the instrument, and fastQC was performed to QC the data. Reads were aligned to the reference sequence with Bowtie, and the resulting bam file was filtered to remove secondary and supplementary alignments and unmapped, and poor-quality reads. Next, for each mapped read, each base aligning to the degenerate part of the template was extracted and added to a pivot table, with each row consisting of a readID and the columns containing corresponding individual bases by position. Each base was labeled as R for reference, N for not called, or the base was left unchanged if it did not match the reference. For each readID, the number of mismatches (bases that were not R or N) was calculated in the region targeted by the LNA. The dataset was then separated by the number of mismatches. For each dataset, the number of reference and non-reference bases were counted per position, allowing base frequencies at each position to be calculated.

## Supplementary Information

Supplementary Information 1.

Supplementary Information 2.

Supplementary Information 3.

## Data Availability

Sequence data is available at SRA with the accession number PRJNA910530. All other data can be found in the manuscript or supplementary files.
