# Supplementary material for: LNA blockers for improved amplification selectivity
Source: Sci Rep. 2023 Mar 24;13:4858. doi: 10.1038/s41598-023-31871-7 (PMC10038989; doi:10.1038/s41598-023-31871-7)
Supplement: Supplementary file 1 — Supplementary Information 1. [file 41598_2023_31871_MOESM1_ESM.pdf]

Supplementary Figure 1: Representative TapeStation results
A representative output of four blockers used with Template_0 is shown. For each lane, the TapeStation calculates the concentration of the amplified DNA that runs ~185 bp. These concentration values are averaged and compared to the values for the No LNA sample run at the same time.

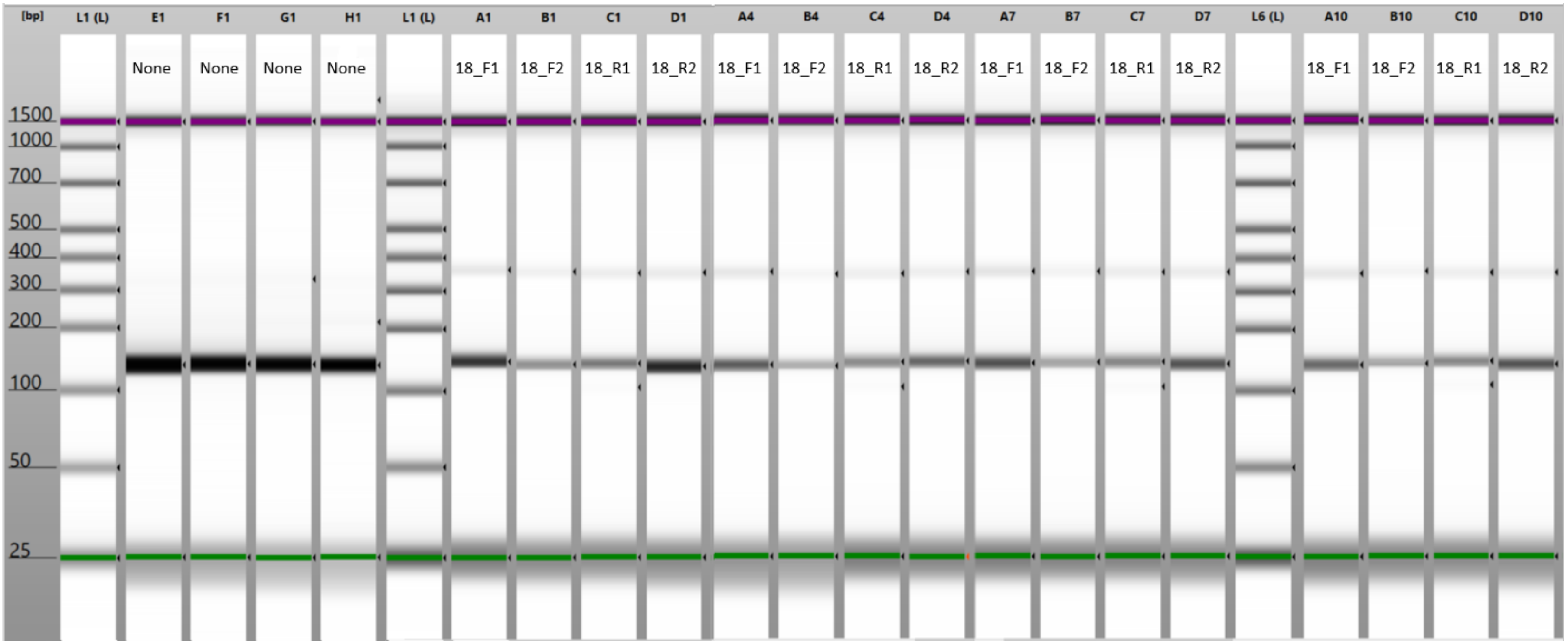

Supplementary Figure 2: Template_D and degenerate regions

The Template_D sequence is shown with the degenerate regions highlighted in red. The primer binding regions are not degenerate. The degenerate regions are synthesized with the base shown in 79% of DNA molecules and with the other three bases in equal amounts for the other 21% of DNA molecules. Because each synthetic step randomly adds this combination of bases, different molecules will have different numbers of sequence variants. Some DNAs will have the exact sequence listed with no variation, while most will have a range of variants relative to the sequence shown. The random distribution of the number of variants in each molecule is shown in Figure 3A. While Template_D is degenerate, the primers and blockers each have a distinct, unique sequence. Primers used to amplify the DNA are in blue, and the LNA blockers used with this template are in purple. Template_D does not have the same sequence as Template_0 because we found it difficult to purchase good quality, highly degenerate oligonucleotides and used the best one available, Template_D, even though it did not match the Template_0 sequence beyond the 20 nt binding region. Though a sequence identical to Template_0 would have been preferred, the large number of degenerate sites affects HPLC purification and lowers the quality of many long oligos.

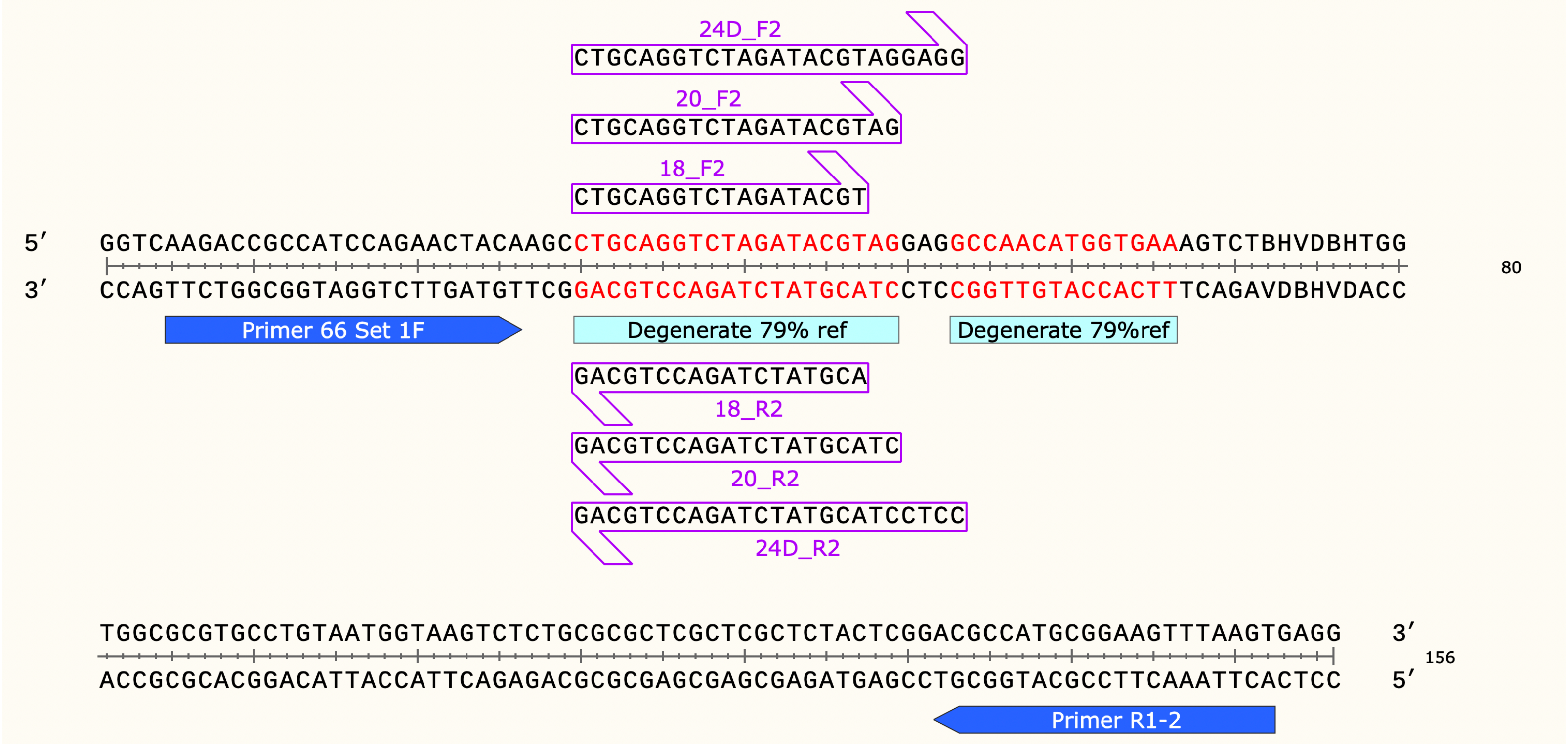

Supplementary Figure 3: Template_A1 and blockers

The sequence of Template_A1 is shown with the changes made relative to Template_0 highlighted in red. Primers used to amplify the DNA are shown in blue, and the LNA blockers used with this template are shown in purple.

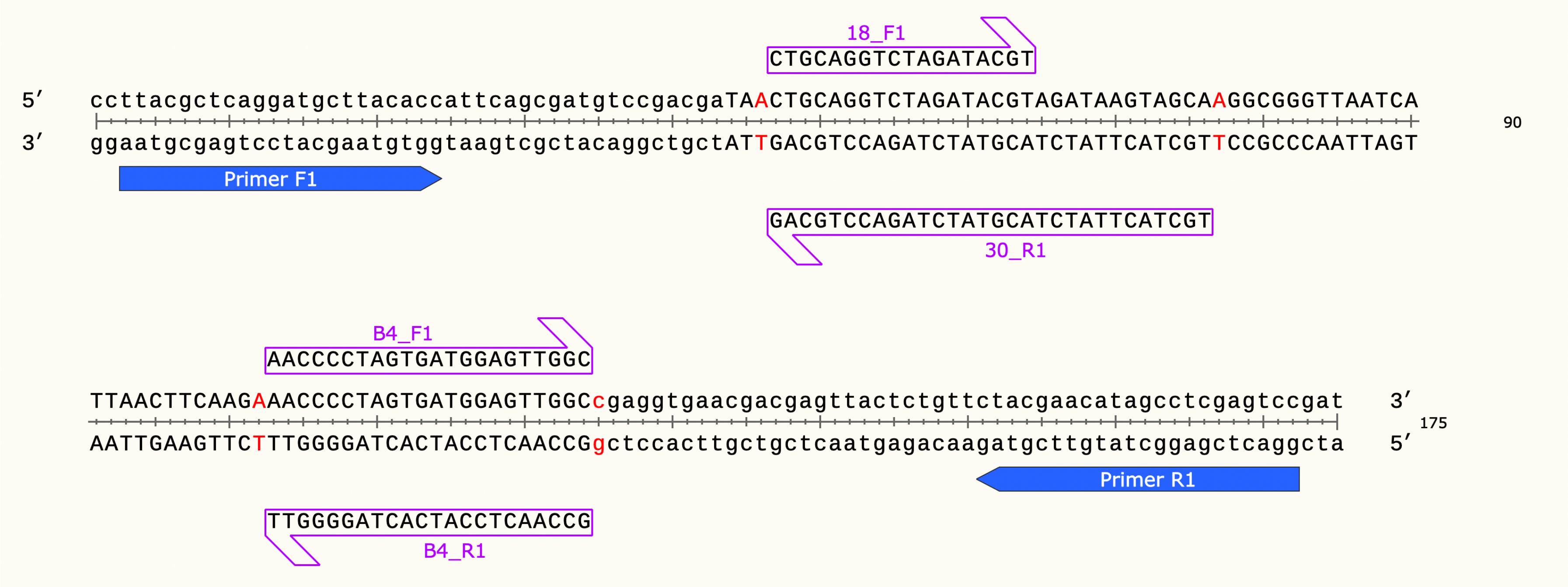

Supplementary Figure 4: Template_A2 and blockers

The sequence of Template_A2 is shown with the changes made relative to Template_0 highlighted in red. Primers used to amplify the DNA are shown in blue, and the LNA blockers used with this template are shown in purple.

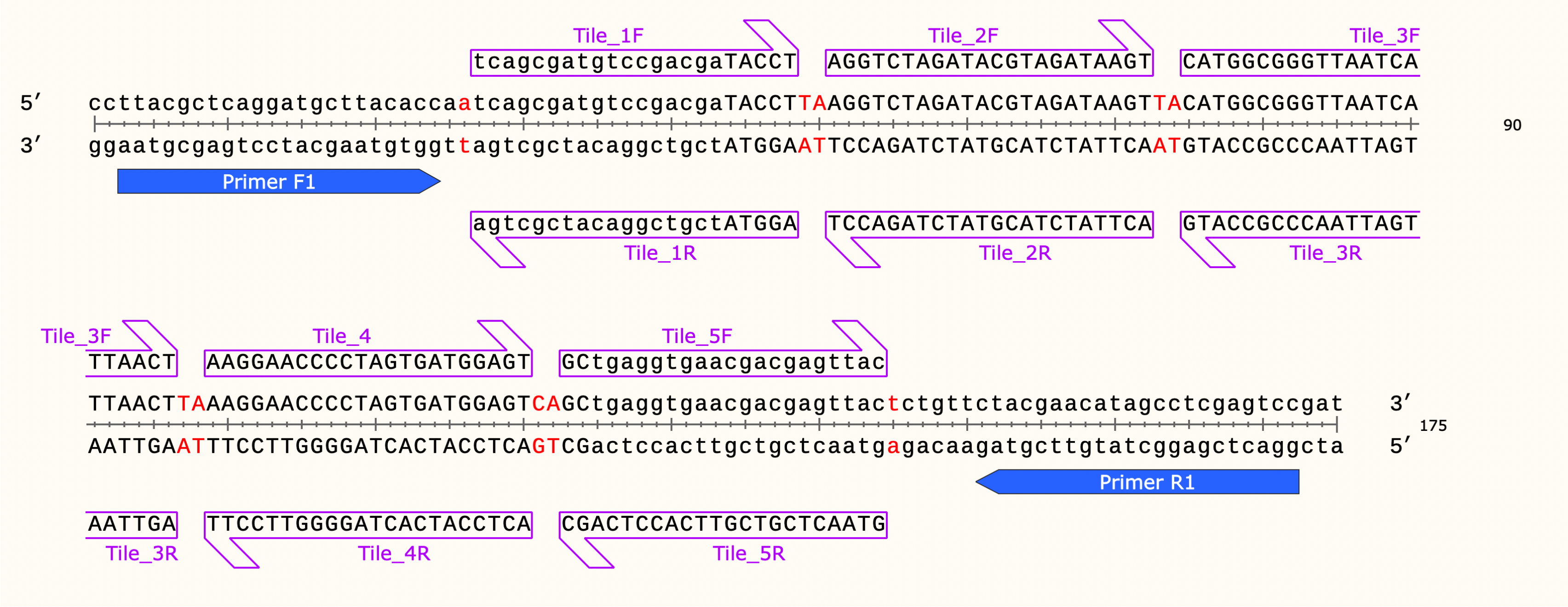

Supplementary Table 1: DNA and LNA sequences

Each DNA and LNA used is listed along with predicted T_m, length, number of LNAs, starting and ending points on the relevant templates, and the sequence. LNA bases are preceded by "+". T_ms were calculated using default conditions at https://www.idtdna.com/calc/analyzer.

DNA/LNA Chimeras	Pred Tm	Length	# LNAs	Oligo Start	Oligo End	Intended Target	Sequence
18_F1	65.6	18	9	47	64	Template_0	/5AmMC6/+CT+GC+AG+GT+CT+AG+AT+AC+GT/3AmMO/
18_F2	68.7	18	9	47	64	Template_0	/5AmMC6/C+TG+CA+GG+TC+TA+GA+TA+CG+T/3AmMO/
18_R1	64.8	18	9	64	47	Template_0	/5AmMC6/+AC+GT+AT+CT+AG+AC+CT+GC+AG/3AmMO/
18_R2	70.9	18	9	64	47	Template_0	/5AmMC6/A+CG+TA+TC+TA+GA+CC+TG+CA+G/3AmMO/
20_F1	70.8	20	10	47	66	Template_0	/5AmMC6/+CT+GC+AG+GT+CT+AG+AT+AC+GT+AG/3AmMO/
20_F2	74.1	20	10	47	66	Template_0	/5AmMC6/C+TG+CA+GG+TC+TA+GA+TA+CG+TA+G/3AmMO/
20_R1	67.5	20	10	66	47	Template_0	/5AmMC6/+CT+AC+GT+AT+CT+AG+AC+CT+GC+AG/3AmMO/
20_R2	71.6	20	10	66	47	Template_0	/5AmMC6/C+TA+CG+TA+TC+TA+GA+CC+TG+CA+G/3AmMO/
22_F1	69.6	22	11	47	68	Template_0	/5AmMC6/+CT+GC+AG+GT+CT+AG+AT+AC+GT+AG+AT/3AmMO/
22_F2	72.8	22	11	47	68	Template_0	/5AmMC6/C+TG+CA+GG+TC+TA+GA+TA+CG+TA+GA+T/3AmMO/
22_R1	69.4	22	11	68	47	Template_0	/5AmMC6/+AT+CT+AC+GT+AT+CT+AG+AC+CT+GC+AG/3AmMO/
22_R2	74.3	22	11	68	47	Template_0	/5AmMC6/A+TC+TA+CG+TA+TC+TA+GA+CC+TG+CA+G/3AmMO/
24D_F2	78.2	24	12	30 (D)	53 (D)	Template_D	/5AmMC6/C+TG+CA+GG+TC+TA+GA+TA+CG+TA+GG+AG+G/3AmMO/
24D_R2	80.8	24	12	53 (D)	30 (D)	Template_D	/5AmMC6/C+CT+CC+TA+CG+TA+TC+TA+GA+CC+TG+CA+G/3AmMO/
24_F1	69.1	24	12	47	70	Template_0	/5AmMC6/+CT+GC+AG+GT+CT+AG+AT+AC+GT+AG+AT+AA/3AmMO/
24_F2	73.1	24	12	47	70	Template_0	/5AmMC6/C+TG+CA+GG+TC+TA+GA+TA+CG+TA+GA+TA+A/3AmMO/
24_R1	69.0	24	12	70	47	Template_0	/5AmMC6/+TT+AT+CT+AC+GT+AT+CT+AG+AC+CT+GC+AG/3AmMO/
24_R2	74.7	24	12	70	47	Template_0	/5AmMC6/T+TA+TC+TA+CG+TA+TC+TA+GA+CC+TG+CA+G/3AmMO/
24D_F2	78.2	24	12	47	NA	Template_0	/5AmMC6/C+TG+CA+GG+TC+TA+GA+TA+CG+TA+GG+AG+G/3AmMO/
24D_R2	80.8	24	12	47	NA	Template_0	/5AmMC6/C+CT+CC+TA+CG+TA+TC+TA+GA+CC+TG+CA+G/3AmMO/
30_F1	75.8	30	15	47	76	Template_0	/5AmMC6/+CT+GC+AG+GT+CT+AG+AT+AC+GT+AG+AT+AA+GT+AG+CA/3AmMO/
30_F2	78.4	30	15	47	76	Template_0	/5AmMC6/C+TG+CA+GG+TC+TA+GA+TA+CG+TA+GA+TA+AG+TA+GC+A/3AmMO/
30_R1	75.5	30	15	76	47	Template_0	/5AmMC6/+TG+CT+AC+TT+AT+CT+AC+GT+AT+CT+AG+AC+CT+GC+AG/3AmMO/
30_R2	81.1	30	15	76	47	Template_0	/5AmMC6/T+GC+TA+CT+TA+TC+TA+CG+TA+TC+TA+GA+CC+TG+CA+G/3AmMO/
B4_F1	79.5	22	11	103	124	Template_0	/5AmMC6/+AA+CC+CC+TA+GT+GA+TG+GA+GT+TG+GC/3AmMO/
B4_F2	80.3	22	11	103	124	Template_0	/5AmMC6/A+AC+CC+CT+AG+TG+AT+GG+AG+TT+GG+C/3AmMO/
B4_R1	76.8	22	11	124	103	Template_0	/5AmMC6/+GC+CA+AC+TC+CA+TC+AC+TA+GG+GG+TT/3AmMO/
B4_R2	81.2	22	11	124	103	Template_0	/5AmMC6/G+CC+AA+CT+CC+AT+CA+CT+AG+GG+GT+T/3AmMO/
Tile_1F1	80.1	22	11	27	48	Template_0	/5AmMC6/+TC+AG+CG+AT+GT+CC+GA+CG+AT+AC+CT/3AmMO/
Tile_1R1	77.4	22	11	48	27	Template_0	/5AmMC6/+AG+GT+AT+CG+TC+GG+AC+AT+CG+CT+GA/3AmMO/
Tile_2F1	64.1	22	11	51	72	Template_0	/5AmMC6/+AG+GT+CT+AG+AT+AC+GT+AG+AT+AA+GT/3AmMO/
Tile_2R1	64.5	22	11	72	51	Template_0	/5AmMC6/+AC+TT+AT+CT+AC+GT+AT+CT+AG+AC+CT/3AmMO/
Tile_3F1	71.3	22	11	75	96	Template_0	/5AmMC6/+CA+TG+GC+GG+GT+TA+AT+CA+TT+AA+CT/3AmMO/
Tile_3R1	70.8	22	11	96	75	Template_0	/5AmMC6/+AG+TT+AA+TG+AT+TA+AC+CC+GC+CA+TG/3AmMO/
Tile_4F1	77.7	22	11	99	120	Template_0	/5AmMC6/+AA+GG+AA+CC+CC+TA+GT+GA+TG+GA+GT/3AmMO/
Tile_4R1	74.6	22	11	120	99	Template_0	/5AmMC6/+AC+TC+CA+TC+AC+TA+GG+GG+TT+CC+TT/3AmMO/
Tile_5F1	75.0	22	11	123	144	Template_0	/5AmMC6/+GC+TG+AG+GT+GA+AC+GA+CG+AG+TT+AC/3AmMO/
Tile_5R1	75.0	22	11	144	123	Template_0	/5AmMC6/+GT+AA+CT+CG+TC+GT+TC+AC+CT+CA+GC/3AmMO/
A1AT_F1_2	67.3	17	8	chr14:94378610	chr14:94378594	A1AT	/5AmMC6/G+AG+AA+AG+GG+AC+TG+AA+GC/3AmMO/
A1AT_F3_2	67.4	16	8	chr14:94378612	chr14:94378597	A1AT	/5AmMC6/A+CG+AG+AA+AG+GG+AC+TG+A/3AmMO/
A1AT_F5_2	67.4	16	8	chr14:94378614	chr14:94378599	A1AT	/5AmMC6/C+GA+CG+AG+AA+AG+GG+AC+T/3AmMO/
A1AT_F7_2	67.9	16	8	chr14:94378616	chr14:94378601	A1AT	/5AmMC6/A+TC+GA+CG+AG+AA+AG+GG+A/3AmMO/
A1AT_R1_2	69.0	16	8	chr14:94378610	chr14:94378625	A1AT	/5AmMC6/C+GT+CG+AT+GG+TC+AG+CA+C/3AmMO/
A1AT_R3_2	66.6	16	8	chr14:94378608	chr14:94378623	A1AT	/5AmMC6/C+TC+GT+CG+AT+GG+TC+AG+C/3AmMO/
A1AT_R5_2	67.0	17	8	chr14:94378606	chr14:94378622	A1AT	/5AmMC6/T+TC+TC+GT+CG+AT+GG+TC+AG/3AmMO/
A1AT_R7_2	64.5	17	8	chr14:94378604	chr14:94378620	A1AT	/5AmMC6/C+TT+TC+TC+GT+CG+AT+GG+TC/3AmMO/
Primers	Pred Tm	Length	# LNAs	Oligo Start	Oligo End	Intended Target	Sequence
F1	56.9	22	0	3	24	Template_0	TTACGCTCAGGATGCTTACACC
R1	56.6	22	0	172	151	Template_0	GGACTCGAGGCTATGTTCGTAG
F3	55.9	22	0	7	28	Template_0	GCTCAGGATGCTTACACCATTC
R3a	56.0	26	0	114	89	Template_0	TCACTAGGGGTTCCTTGTAGTTAATG
R3b	56.3	21	0	127	107	Template_0	TCAGCCAACTCCATCACTAGG
66 Set 1	57.5	22	0	5 (D)	26 (D)	Template_D	AAGACCGCCATCCAGAACTACA
R1-2	57.4	21	0	153 (D)	133 (D)	Template_D	ACTTAAACTTCCGCATGGCGT
MR19_F2	54.3	21	0	chr14:94379165	chr14:94379145	A1AT	ACAGAAAGGTTTGGGCTAAGT
A1ATR_R1	54.0	23	0	chr14:94378520	chr14:94378542	A1AT	AGACAAAGGGTTTGTTGAACTTG
Templates	Pred Tm	Length	# LNAs	Oligo Start	Oligo End		Sequence
Template_0	NA	175	0	1	175		CCTTACGCTCAGGATGCTTACACCATTCAGCGATGTCCGACGATACCTGCAGGTCTAGATACGTAGATAAGTAGCATGGCGGGTTAATCATTAACTACAAGGAACCCCTAGTGATGGAGTTGGCTGAGGTGAACGACGAGTTACGCTGTTCTACGAACATAGCCTCGAGTCCGAT
Template_A1	NA	175	0	1	175		CCTTACGCTCAGGATGCTTACACCATTCAGCGATGTCCGACGATAACTGCAGGTCTAGATACGTAGATAAGTAGCAAGGCGGGTTAATCATTAACTTCAAGAAACCCCTAGTGATGGAGTTGGCCGAGGTGAACGACGAGTTACTCTGTTCTACGAACATAGCCTCGAGTCCGAT
Template_A2	NA	175	0	1	175		CCTTACGCTCAGGATGCTTACACCAATCAGCGATGTCCGACGATACCTTAAGGTCTAGATACGTAGATAAGTTACATGGCGGGTTAATCATTAACTTAAAGGAACCCCTAGTGATGGAGTCAGCTGAGGTGAACGACGAGTTACTCTGTTCTACGAACATAGCCTCGAGTCCGAT
Template_D	NA	156	0	1	156		GGTCAAGACCGCCATCCAGAACTACAAGCCTGCAGGTCTAGATACGTAGGAGGCCAACATGGTGAAAGTCTBHVDBHTGGTGGCGCGTGCCTGTAATGGTAAGTCTCTGCGCGCTCGCTCGCTCTACTCGGACGCCATGCGGAAGTTTAAGTGAGG
Template_U	NA	225	0	1	225		CCTTACGCTCAGGATGCTTACACCATTCAGCGATGTCCGACGATGGAGTTCCTGATGTATCCCTATACTAATGGCGATGAAATCAATGGCGACCTATACGGATAATAGATTTCTGCCGAGCCTGCCCAGTGCCTCGAACACCAGTGCCTCGGAACTTAACGGGTTGTAGCCGATTGAGGTGAACGACGAGTTACGCTGTTCTACGAACATAGCCTCGAGTCCGAT

Supplementary Table 2: Predicted and observed mismatch frequencies in unblocked samples
The mismatch frequencies calculated from the permutation equation listed in the text and graphed in Figure 3A are shown relative to the observed frequencies for samples sequenced with no LNA blockers.

	18 nt		20 nt		21 nt	
Mismatches	Predicted	No LNA	Predicted	No LNA	Predicted	No LNA
0	1.44	1.37	0.90	0.83	0.71	0.74
1	6.87	6.26	4.77	4.28	3.95	3.96
2	15.53	14.13	12.04	10.64	10.51	10.24
3	22.02	20.57	19.20	17.47	17.69	17.50
4	21.95	21.59	21.69	20.89	21.16	21.24
5	16.34	17.17	18.45	18.89	19.13	19.46
6	9.41	10.80	12.26	13.68	13.56	13.85
7	4.29	5.25	6.52	7.89	7.72	7.83
8	1.57	2.06	2.82	3.62	3.59	3.52
9	0.46	0.64	1.00	1.37	1.38	1.29
10	0.11	0.16	0.29	0.44	0.44	0.36

Supplementary Table 3: Mismatch read frequency by base
The raw number of sequence reads with the given lengths and blockers are provided for each base by position within the degenerate regions being blocked. At the end of the table, the percentage of total reads for 1 and 2 mismatch reads are provided for each.

No_LNA_18	1	2	3	4	5	6	7	8	9	10	11	12	13	14	15	16	17	18
No_LNA_18_0mm_count																		
R	4609	4609	4609	4609	4609	4609	4609	4609	4609	4609	4609	4609	4609	4609	4609	4609	4609	4609
T	0	0	0	0	0	0	0	0	0	0	0	0	0	0	0	0	0	0
A	0	0	0	0	0	0	0	0	0	0	0	0	0	0	0	0	0	0
C	0	0	0	0	0	0	0	0	0	0	0	0	0	0	0	0	0	0
G	0	0	0	0	0	0	0	0	0	0	0	0	0	0	0	0	0	0
No_LNA_18_1mm_count																		
R	19494	20154	20248	19955	19414	20236	20460	19722	20275	19723	19453	20041	19573	19771	19518	20257	20190	19587
T	364	0	257	305	466	282	193	0	280	0	415	360	398	0	484	286	256	0
A	713	318	258	156	0	206	158	252	228	295	0	261	0	294	0	240	222	331
C	0	207	300	0	580	339	252	581	0	535	661	401	560	552	607	0	395	562
G	492	384	0	647	603	0	0	508	280	510	534	0	532	446	454	280	0	583
No_LNA_18_2mm_count																		
R	41041	43506	43421	42340	40170	43959	44465	41627	43710	41573	40117	42961	40748	41543	40770	43952	43654	40891
T	1618	0	1373	1289	2070	1236	1025	0	1279	0	1946	1499	1921	0	2195	1210	1252	0
A	2858	1221	1280	826	0	860	693	1095	1080	1458	0	1205	0	1459	0	995	937	1539
C	0	1095	1454	0	2662	1473	1345	2531	0	2235	2960	1863	2617	2426	2669	0	1685	2647
G	2011	1706	0	3073	2626	0	0	2275	1459	2262	2505	0	2242	2100	1894	1371	0	2451
No_LNA_18_3mm_count																		
R	55146	59900	59724	57450	53826	61201	61988	56255	60702	56337	53700	59160	54490	56408	54657	61141	60603	55477
T	3770	0	3063	2862	4242	2785	2571	0	2908	0	4077	3313	4108	0	4621	2737	2788	0
A	6003	2713	2992	2011	0	1919	1655	2525	2477	3145	0	2678	0	3062	0	2321	2139	3189
C	0	2715	3432	0	5646	3306	2997	5375	0	5029	6057	4060	5695	5227	5572	0	3681	5665
G	4292	3883	0	6888	5497	0	0	5056	3124	4700	5377	0	4918	4514	4361	3012	0	4880
No_LNA_18_4mm_count																		
R	52506	58501	58632	55596	51320	61339	62680	54908	60404	54804	51636	59019	52563	55024	52618	60998	60879	53827
T	5698	0	4576	4113	5991	3962	3379	0	4076	0	5678	4572	5740	0	6450	3940	3898	0
A	8520	3878	4299	2987	0	2741	2291	3439	3530	4424	0	3572	0	4214	0	3354	2760	4343
C	0	4582	5154	0	7826	4619	4311	7600	0	6928	8156	5498	7657	7281	7610	0	5124	7673
G	5937	5700	0	9965	7524	0	0	6714	4651	6505	7191	0	6701	6142	5983	4369	0	6818
No_LNA_18_5mm_count																		
R	37645	42638	43184	40936	37101	46314	47644	40547	45111	40193	37323	44088	38102	40898	38429	46079	45748	39134
T	6030	0	4777	4040	5817	4022	3530	0	4191	0	5581	4587	5457	0	6050	3948	3920	0
A	8421	3940	4502	2948	0	2702	2250	3203	3632	4422	0	3607	0	4115	0	3441	2901	4391
C	0	5246	5315	0	7816	4740	4354	7553	0	6815	7977	5496	7548	6788	7397	0	5209	7612
G	5682	5954	0	9854	7044	0	0	6475	4844	6348	6897	0	6671	5977	5902	4310	0	6641
No_LNA_18_6mm_count																		
R	20940	24445	24937	23373	20984	27689	28533	23211	26709	23345	21516	26034	21988	23488	21936	27258	27284	22494
T	4618	0	3696	3072	4375	2965	2733	0	3163	0	4023	3424	3960	0	4532	3154	2855	0
A	6390	3007	3518	2326	0	2109	1815	2517	2817	3166	0	2750	0	3054	0	2568	2155	3343
C	0	4364	4196	0	5832	3584	3266	5715	0	5090	5756	4139	5643	5221	5448	0	4053	5720
G	4399	4531	0	7576	5156	0	0	4904	3658	4746	5052	0	4756	4584	4431	3367	0	4790
No_LNA_18_7mm_count																		
R	8916	10604	11047	10073	9131	12600	13073	10444	12136	10333	9423	11926	9557	10478	9780	12417	12479	9975
T	2788	0	2160	1900	2453	1758	1617	0	1758	0	2252	1987	2265	0	2410	1817	1698	0
A	3608	1754	2100	1269	0	1198	1012	1444	1678	1794	0	1422	0	1634	0	1468	1239	1825
C	0	2645	2365	0	3212	2116	1970	3155	0	2893	3152	2337	3169	2983	3021	0	2256	3239
G	2360	2669	0	4430	2876	0	0	2629	2100	2652	2845	0	2681	2577	2461	1970	0	2633
No_LNA_18_8mm_count																		
R	3035	3713	3918	3607	3214	4705	4930	3638	4399	3644	3267	4337	3297	3723	3370	4469	4613	3431
T	1217	0	988	730	1062	807	653	0	807	0	998	891	966	0	1071	869	697	0
A	1652	766	983	583	0	493	444	638	700	807	0	612	0	695	0	669	556	814
C	0	1224	1042	0	1419	926	904	1472	0	1282	1431	1091	1393	1364	1328	0	1065	1441
G	1027	1228	0	2011	1236	0	0	1183	1025	1198	1235	0	1275	1149	1162	924	0	1245
No_LNA_18_9mm_count																		
R	775	1000	1102	1014	873	1367	1458	988	1290	978	851	1248	898	1033	915	1263	1346	915
T	418	0	348	241	382	253	210	0	252	0	346	307	322	0	340	280	269	0
A	590	269	341	196	0	167	156	217	259	300	0	212	0	224	0	245	186	314
C	0	435	355	0	511	359	322	508	0	434	491	379	486	471	480	0	345	513
G	363	442	0	695	380	0	0	433	345	434	458	0	440	418	411	358	0	404
No_LNA_18_10mm_count																		
R	132	200	225	218	185	298	348	225	282	208	209	278	184	238	210	270	307	191
T	139	0	111	84	99	64	51	0	62	0	69	66	98	0	88	84	66	0
A	165	85	98	53	0	55	39	69	72	82	0	71	0	54	0	66	47	77
G	90	126	0	171	106	0	0	103	110	121	126	0	102	93	109	106	0	92
C	0	115	92	0	136	109	88	129	0	115	122	111	142	141	119	0	106	166

F2 18	1	2	3	4	5	6	7	8	9	10	11	12	13	14	15	16	17	18
F2_18_0mm_count																		
R	2762	2762	2762	2762	2762	2762	2762	2762	2762	2762	2762	2762	2762	2762	2762	2762	2762	2762
T	0	0	0	0	0	0	0	0	0	0	0	0	0	0	0	0	0	0
A	0	0	0	0	0	0	0	0	0	0	0	0	0	0	0	0	0	0
C	0	0	0	0	0	0	0	0	0	0	0	0	0	0	0	0	0	0
G	0	0	0	0	0	0	0	0	0	0	0	0	0	0	0	0	0	0
F2_18_1mm_count																		
R	27229	27741	27309	26671	25449	26999	27375	26612	26746	26600	25533	26666	26220	26454	26034	26991	27825	27343
T	227	0	350	482	773	490	391	0	547	0	724	617	584	0	816	445	159	0
A	524	181	308	353	0	230	172	298	439	365	0	331	0	462	0	382	122	208
C	0	127	374	0	1053	622	403	615	0	521	1090	727	680	603	794	0	235	375
G	361	292	0	835	1066	0	0	816	609	855	994	0	857	822	697	523	0	415
F2_18_2mm_count																		
R	85117	88207	86837	84987	78647	86758	88081	83307	86215	82621	78891	84629	80642	82587	80390	87298	88431	84979
T	2301	0	2701	2786	4603	2925	2549	0	3023	0	4125	3533	4023	0	4923	2614	2149	0
A	4268	2037	2425	1926	0	1753	1465	2228	2473	3037	0	2593	0	3000	0	2157	1587	2245
C	0	1603	2951	0	5831	3478	2819	4554	0	4526	6350	4159	5169	4779	5374	0	2747	3849
G	3228	3067	0	5215	5833	0	0	4825	3203	4730	5548	0	5080	4548	4227	2845	0	3841
F2_18_3mm_count																		
R	128217	136778	135778	131577	120625	137734	140217	128897	136577	127287	121078	133301	123580	128303	123769	138714	138226	128377
T	7690	0	7065	6408	10297	6819	6141	0	7002	0	9672	7884	9681	0	10898	6478	6180	0
A	12294	5964	6531	4668	0	4545	3831	5311	5936	7440	0	6374	0	7108	0	5317	4636	6620
C	0	5701	7895	0	13427	8171	7080	11796	0	11561	14202	9710	12523	11733	12722	0	8227	11553
G	9068	8826	0	14616	12920	0	0	11265	7754	10981	12317	0	11485	10125	9880	6760	0	10719
F2_18_4mm_count																		
R	125531	137379	137481	132058	120075	142776	146090	129959	140733	128674	120851	137441	123457	129121	123895	143519	141944	127430
T	12827	0	10933	9474	14288	9564	8582	0	10006	0	13468	10991	13398	0	14863	9370	9256	0
A	18866	9033	9993	6942	0	6787	5557	7682	8694	10502	0	8833	0	10150	0	7724	6933	10027
C	0	10783	12194	0	18744	11474	10372	17490	0	16274	19262	13336	17912	16898	17767	0	12468	17612
G	13377	13406	0	22127	17494	0	0	15470	11168	15151	17020	0	15834	14432	14076	9988	0	15532
F2_18_5mm_count																		
R	90122	100563	101826	96488	87791	108957	111516	96208	106779	94905	88430	103745	90400	95746	90838	108970	107659	92998
T	13904	0	11332	9643	13827	9553	8859	0	9851	0	13037	11085	12852	0	14483	9395	9376	0
A	18847	9413	10542	6987	0	6564	5642	7790	8559	10497	0	8706	0	9849	0	7857	6820	10187
C	0	12595	12757	0	18317	11383	10440	17435	0	16056	18435	12921	17421	16480	17170	0	12602	17798
G	13584	13886	0	23339	16522	0	0	15024	11268	14999	16555	0	15784	14382	13966	10235	0	15474
F2_18_6mm_count																		
R	48846	55765	57239	54062	48324	63036	65274	54227	61244	53356	49415	59800	50399	54130	50634	62716	62260	52113
T	10708	0	8498	7095	10046	7128	6225	0	7311	0	9412	7959	9179	0	10370	7163	6902	0
A	14177	7026	8308	5164	0	4844	4136	5495	6474	7370	0	6206	0	7034	0	5943	5085	7490
C	0	10124	9525	0	13431	8562	7935	12870	0	11923	12928	9605	12733	11998	12320	0	9323	12782
G	9839	10655	0	17249	11769	0	0	10978	8541	10921	11815	0	11259	10408	10246	7748	0	11185
F2_18_7mm_count																		
R	20962	24510	25574	24154	21277	29190	30404	24294	28143	23858	21920	27644	22042	24203	22680	28880	28960	22756
T	6325	0	5151	4077	5575	4145	3614	0	4125	0	5294	4648	5332	0	5646	4192	3838	0
A	8183	4063	4892	2929	0	2718	2454	3194	3686	4215	0	3295	0	3833	0	3372	2900	4329
C	0	6246	5424	0	7569	4988	4569	7393	0	6785	7290	5454	7293	6870	6978	0	5343	7545
G	5571	6222	0	9881	6620	0	0	6160	5087	6183	6537	0	6374	6135	5737	4597	0	6411
F2_18_8mm_count																		
R	6739	8199	8603	8036	7293	10493	10949	8345	9877	8188	7425	9793	7464	8313	7583	10331	10240	7719
T	2831	0	2375	1751	2360	1789	1546	0	1774	0	2200	1984	2146	0	2411	1793	1718	0
A	3532	1761	2135	1274	0	1193	1062	1348	1659	1804	0	1399	0	1592	0	1421	1240	1918
C	0	2830	2446	0	3168	2084	2002	3180	0	2889	3177	2383	3162	2947	3012	0	2361	3260
G	2457	2769	0	4498	2738	0	0	2686	2249	2678	2757	0	2787	2707	2553	2014	0	2662
F2_18_9mm_count																		
R	1643	2043	2323	2153	1852	2904	3074	2256	2703	2169	1965	2761	1864	2172	2028	2809	2845	1971
T	977	0	770	579	806	589	502	0	584	0	745	591	729	0	780	612	581	0
A	1239	603	738	394	0	390	320	431	568	614	0	463	0	543	0	469	407	645
C	0	1049	784	0	1058	732	719	1055	0	940	985	800	1097	980	985	0	782	1133
G	756	920	0	1489	899	0	0	873	760	892	920	0	925	920	822	725	0	866
F2_18_10mm_count																		
R	303	382	444	413	364	564	634	425	573	390	376	580	352	444	363	560	588	333
T	229	0	189	135	193	136	124	0	125	0	165	154	178	0	197	150	133	0
A	302	168	174	109	0	111	89	110	123	144	0	102	0	122	0	121	101	172
C	0	232	204	0	232	200	164	278	0	262	264	175	242	222	242	0	189	287
G	177	229	0	354	222	0	0	198	190	215	206	0	239	223	209	180	0	219

R2_18	1	2	3	4	5	6	7	8	9	10	11	12	13	14	15	16	17	18
R2_18_0mm_count																		
R	2170	2170	2170	2170	2170	2170	2170	2170	2170	2170	2170	2170	2170	2170	2170	2170	2170	2170
T	0	0	0	0	0	0	0	0	0	0	0	0	0	0	0	0	0	0
A	0	0	0	0	0	0	0	0	0	0	0	0	0	0	0	0	0	0
C	0	0	0	0	0	0	0	0	0	0	0	0	0	0	0	0	0	0
G	0	0	0	0	0	0	0	0	0	0	0	0	0	0	0	0	0	0
R2_18_1mm_count																		
R	20447	20706	20530	20499	19784	20319	20515	20059	20459	19841	19508	20053	19555	19684	19395	20485	20482	20238
T	214	0	273	230	416	343	261	0	276	0	476	438	498	0	611	282	246	0
A	370	207	271	157	0	230	171	254	258	373	0	315	0	388	0	227	209	240
C	0	126	253	0	621	435	380	558	0	577	785	521	719	698	777	0	390	489
G	296	288	0	441	506	0	0	456	334	536	558	0	555	557	544	333	0	360
R2_18_2mm_count																		
R	52539	54972	54322	53373	50317	54373	54937	52154	54357	51660	50151	53151	50567	51748	50389	54819	54384	52123
T	1673	0	1650	1521	2567	1703	1519	0	1780	0	2406	2125	2469	0	2973	1547	1653	0
A	2977	1309	1568	1081	0	1217	1057	1308	1486	1948	0	1594	0	1872	0	1343	1222	1711
C	0	1108	1856	0	3262	2103	1883	3141	0	2977	3747	2526	3452	3227	3421	0	2137	2908
G	2207	2007	0	3421	3250	0	0	2793	1773	2811	3092	0	2908	2549	2613	1687	0	2654
R2_18_3mm_count																		
R	74655	80193	79772	77398	71923	81346	82808	75689	80791	75217	71925	79106	73054	75442	73133	81711	80922	74965
T	4741	0	4220	3711	5892	3977	3465	0	4075	0	5536	4478	5567	0	6198	3719	3818	0
A	7720	3598	4021	2744	0	2693	2295	3266	3443	4374	0	3572	0	4331	0	3180	2819	4158
C	0	3644	4657	0	7661	4654	4102	7248	0	6784	8148	5514	7437	7000	7598	0	5111	7055
G	5554	5235	0	8817	7194	0	0	6467	4361	6295	7061	0	6612	5897	5741	4060	0	6492
R2_18_4mm_count																		
R	72325	79543	79625	76499	70101	83154	84789	75238	81971	74744	70782	79958	71773	75209	72056	83389	82497	73831
T	7643	0	6337	5463	8306	5605	4997	0	5713	0	7722	6330	7709	0	8510	5424	5343	0
A	11224	5400	6100	4134	0	3712	3220	4665	5015	6072	0	5000	0	5928	0	4456	4027	6058
C	0	6335	7044	0	10850	6635	6100	10219	0	9487	11021	7818	10486	9775	10374	0	7239	10244
G	7914	7828	0	13010	9849	0	0	8984	6407	8803	9581	0	9138	8194	8166	5837	0	8973
R2_18_5mm_count																		
R	51809	58184	58899	55753	50811	63111	64660	55680	61823	55247	51757	60357	52337	55768	52828	62923	62466	54342
T	8007	0	6536	5784	7992	5565	5033	0	5740	0	7282	6286	7647	0	8401	5627	5325	0
A	11333	5557	6356	4143	0	3859	3351	4431	5189	5962	0	4937	0	5632	0	4631	4123	5918
C	0	7302	7344	0	10664	6600	6091	10228	0	9404	10574	7555	10220	9597	10016	0	7221	10098
G	7986	8092	0	13455	9668	0	0	8796	6383	8522	9522	0	8931	8138	7890	5954	0	8777
R2_18_6mm_count																		
R	28857	32883	33391	31324	28475	37072	38272	31893	35973	31443	29249	35332	29676	31953	29956	36960	36597	30686
T	6189	0	5210	4289	5959	4171	3851	0	4268	0	5510	4639	5519	0	6157	4282	4086	0
A	8555	4210	4779	3074	0	2820	2480	3326	3823	4389	0	3550	0	4171	0	3474	2983	4519
C	0	5802	5786	0	7786	5103	4563	7677	0	6860	7644	5645	7447	6973	7273	0	5500	7520
G	5565	6271	0	10479	6946	0	0	6270	5102	6474	6763	0	6524	6069	5780	4450	0	6441
R2_18_7mm_count																		
R	12204	14386	15044	13845	12443	17092	17724	14134	16668	14120	13011	16172	12912	14280	13445	16912	16928	13615
T	3744	0	2942	2431	3295	2497	2184	0	2372	0	2985	2750	3125	0	3313	2496	2359	0
A	4933	2361	2772	1838	0	1643	1450	1925	2084	2468	0	1925	0	2279	0	2033	1630	2490
C	0	3747	3327	0	4475	2853	2727	4422	0	3801	4262	3238	4345	4058	3916	0	3168	4340
G	3204	3591	0	5971	3872	0	0	3604	2961	3696	3827	0	3703	3468	3411	2644	0	3640
R2_18_8mm_count																		
R	3875	4693	5028	4683	4154	6032	6287	4782	5788	4767	4364	5628	4404	4919	4519	5909	5979	4509
T	1633	0	1364	1001	1443	1041	912	0	989	0	1262	1114	1242	0	1421	1080	937	0
A	2157	1030	1265	757	0	700	610	816	926	1133	0	870	0	893	0	891	739	1102
C	0	1682	1375	0	1823	1259	1223	1920	0	1623	1825	1420	1855	1734	1652	0	1377	1846
G	1367	1627	0	2591	1612	0	0	1514	1329	1509	1581	0	1531	1486	1440	1152	0	1575
R2_18_9mm_count																		
R	1023	1278	1458	1353	1200	1831	1877	1355	1628	1321	1211	1664	1216	1415	1233	1716	1789	1253
T	588	0	466	347	479	353	311	0	374	0	460	409	430	0	484	390	349	0
A	764	386	450	265	0	234	229	314	374	377	0	262	0	309	0	316	280	394
C	0	622	495	0	645	451	452	666	0	578	644	534	663	634	638	0	451	651
G	494	583	0	904	545	0	0	534	493	593	554	0	560	511	514	447	0	571
R2_18_10mm_count																		
R	184	239	292	293	255	399	437	287	356	275	253	373	246	299	241	373	365	249
T	171	0	156	110	122	80	79	0	93	0	108	91	107	0	114	103	93	0
A	198	124	100	64	0	77	57	60	102	98	0	68	0	100	0	83	70	100
C	0	159	129	0	167	121	104	190	0	155	152	145	195	156	176	0	149	185
G	124	155	0	210	133	0	0	140	126	149	164	0	129	122	146	118	0	143

No_LNA_20	1	2	3	4	5	6	7	8	9	10	11	12	13	14	15	16	17	18	19	20
No_LNA_20_0mm_count																				
R	2801	2801	2801	2801	2801	2801	2801	2801	2801	2801	2801	2801	2801	2801	2801	2801	2801	2801	2801	2801
T	0	0	0	0	0	0	0	0	0	0	0	0	0	0	0	0	0	0	0	0
A	0	0	0	0	0	0	0	0	0	0	0	0	0	0	0	0	0	0	0	0
C	0	0	0	0	0	0	0	0	0	0	0	0	0	0	0	0	0	0	0	0
G	0	0	0	0	0	0	0	0	0	0	0	0	0	0	0	0	0	0	0	0
No_LNA_20_1mm_count																				
R	13379	13776	13905	13733	13376	13920	14031	13574	13888	13562	13444	13758	13502	13616	13472	13884	13873	13474	13337	13849
T	241	0	154	188	293	165	116	0	182	0	242	223	232	0	290	169	140	0	305	176
A	444	212	152	91	0	114	100	162	140	184	0	156	0	165	0	162	136	217	0	125
C	0	137	176	0	364	188	140	339	0	327	373	250	335	332	359	0	238	337	425	237
G	323	262	0	375	354	0	0	312	177	314	328	0	318	274	266	172	0	359	320	0
No_LNA_20_2mm_count																				
R	31320	33038	33013	32273	30912	33357	33748	31721	33240	31825	30883	32741	31233	31739	31236	33396	33196	31154	30775	32970
T	1091	0	929	866	1350	830	668	0	837	0	1254	1009	1298	0	1448	802	837	0	1329	892
A	1979	844	863	538	0	580	459	741	714	971	0	799	0	961	0	661	617	1042	0	729
C	0	778	960	0	1777	998	890	1730	0	1459	1984	1216	1748	1647	1792	0	1115	1818	1998	1174
G	1375	1105	0	2088	1726	0	0	1573	974	1510	1644	0	1486	1418	1289	906	0	1751	1663	0
No_LNA_20_3mm_count																				
R	48067	51780	51649	49929	46967	52771	53302	49066	52328	48972	46855	51134	47520	49010	47667	52723	52199	47934	46622	51915
T	2811	0	2276	2146	3229	2111	1913	0	2215	0	3143	2513	3065	0	3520	2054	2116	0	3315	2248
A	4612	2014	2233	1475	0	1401	1257	1857	1868	2361	0	2023	0	2342	0	1710	1587	2538	0	1762
C	0	1957	2572	0	4284	2447	2258	4087	0	3805	4621	3060	4390	3965	4295	0	2828	4399	4783	2805
G	3240	2979	0	5180	4250	0	0	3720	2319	3592	4111	0	3755	3413	3248	2243	0	3859	4010	0
No_LNA_20_4mm_count																				
R	52865	58369	58297	55699	51790	60353	61563	54708	59781	54676	51709	58288	52935	54830	52890	60263	59852	53639	51545	59180
T	4803	0	3856	3495	5102	3397	2937	0	3445	0	4917	3917	4915	0	5580	3353	3342	0	5153	3638
A	7322	3362	3698	2542	0	2417	1964	3022	3027	3875	0	3123	0	3646	0	2862	2500	3866	0	2910
C	0	3672	4351	0	6727	4035	3738	6561	0	6058	7210	4874	6612	6378	6547	0	4508	6704	7345	4474
G	5212	4799	0	8466	6583	0	0	5911	3949	5593	6366	0	5740	5348	5185	3724	0	5993	6159	0
No_LNA_20_5mm_count																				
R	43812	49056	49380	46792	42796	52243	53652	46351	51229	46066	43280	50105	43901	46614	44195	52026	51837	45207	43023	50830
T	5718	0	4665	4029	5886	3935	3489	0	4089	0	5522	4466	5554	0	6094	3851	3891	0	5633	4209
A	8296	3831	4290	2924	0	2674	2197	3233	3494	4337	0	3501	0	4144	0	3311	2774	4290	0	3307
C	0	4860	5158	0	7696	4641	4155	7391	0	6742	7840	5421	7426	6773	7410	0	4991	7530	7918	5147
G	5667	5746	0	9748	7115	0	0	6518	4681	6348	6851	0	6612	5962	5794	4305	0	6466	6919	0
No_LNA_20_6mm_count																				
R	28684	32769	33266	31452	28426	36246	37178	31165	35042	31118	28739	34201	29203	31477	29312	35736	35746	30167	28814	35091
T	5231	0	4121	3470	4966	3346	3017	0	3610	0	4669	3954	4675	0	5303	3532	3311	0	4728	3566
A	7167	3366	3921	2569	0	2368	2032	2822	3178	3669	0	3186	0	3440	0	2964	2458	3745	0	2855
C	0	4760	4680	0	6684	4028	3761	6489	0	5754	6764	4647	6494	5882	6286	0	4473	6522	6640	4476
G	4906	5093	0	8497	5912	0	0	5512	4158	5447	5816	0	5616	5189	5087	3756	0	5554	5806	0
No_LNA_20_7mm_count																				
R	14731	17216	17746	16392	14836	19771	20433	16573	19140	16547	15271	18725	15523	16742	15629	19480	19489	16210	15176	18948
T	3615	0	2813	2474	3319	2355	2120	0	2360	0	3071	2650	2970	0	3321	2424	2236	0	3149	2565
A	4900	2333	2746	1765	0	1584	1364	1948	2184	2405	0	1985	0	2322	0	2012	1694	2448	0	1970
C	0	3401	3201	0	4408	2796	2589	4332	0	3925	4304	3146	4341	3980	4159	0	3087	4261	4384	3023
G	3260	3556	0	5875	3943	0	0	3653	2822	3629	3860	0	3672	3462	3397	2590	0	3587	3797	0
No_LNA_20_8mm_count																				
R	5912	7168	7459	6898	6085	8617	8992	6985	8215	7033	6274	8075	6493	7062	6649	8413	8498	6827	6311	8182
T	1969	0	1550	1242	1796	1278	1111	0	1280	0	1644	1402	1564	0	1699	1322	1129	0	1649	1377
A	2620	1217	1521	899	0	834	698	986	1147	1281	0	1004	0	1146	0	1030	869	1241	0	1030
C	0	1877	1649	0	2319	1450	1378	2311	0	2029	2259	1698	2197	2169	2050	0	1683	2259	2237	1590
G	1678	1917	0	3140	1979	0	0	1897	1537	1836	2002	0	1925	1802	1781	1414	0	1852	1982	0
No_LNA_20_9mm_count																				
R	2036	2469	2617	2358	2123	3145	3262	2395	2909	2330	2120	2841	2179	2487	2158	2967	3072	2309	2168	2853
T	814	0	693	511	691	508	409	0	550	0	648	620	673	0	711	569	487	0	665	564
A	1068	536	645	381	0	320	312	424	501	564	0	421	0	457	0	431	359	552	0	473
C	0	819	663	0	960	645	635	980	0	900	995	736	918	878	968	0	700	958	923	728
G	700	794	0	1368	844	0	0	819	658	824	855	0	848	796	781	651	0	799	862	0
No_LNA_20_10mm_count																				
R	528	672	755	690	599	895	1000	685	873	662	603	856	597	676	663	844	949	679	588	816
T	290	0	231	168	258	171	143	0	162	0	223	209	235	0	221	191	161	0	234	192
A	410	188	240	142	0	127	107	165	184	198	0	145	0	150	0	184	119	191	0	173
C	0	297	237	0	332	270	213	315	0	288	332	253	347	362	305	0	234	339	372	282
G	235	306	0	463	274	0	0	298	244	315	305	0	284	275	274	244	0	254	269	0

F2 20	1	2	3	4	5	6	7	8	9	10	11	12	13	14	15	16	17	18	19	20
F2_20_0mm_count																				
R	2636	2636	2636	2636	2636	2636	2636	2636	2636	2636	2636	2636	2636	2636	2636	2636	2636	2636	2636	2636
T	0	0	0	0	0	0	0	0	0	0	0	0	0	0	0	0	0	0	0	0
A	0	0	0	0	0	0	0	0	0	0	0	0	0	0	0	0	0	0	0	0
C	0	0	0	0	0	0	0	0	0	0	0	0	0	0	0	0	0	0	0	0
G	0	0	0	0	0	0	0	0	0	0	0	0	0	0	0	0	0	0	0	0
F2_20_1mm_count																				
R	23276	23800	23678	23096	21899	23307	23710	23122	23152	23131	22450	23121	22835	22966	22755	23264	23663	23161	23177	23904
T	238	0	243	389	688	436	272	0	407	0	500	446	408	0	595	376	260	0	339	153
A	515	184	207	245	0	176	108	236	359	262	0	249	0	334	0	307	143	234	0	141
C	0	96	265	0	851	474	303	391	0	356	718	577	534	473	572	0	327	467	454	195
G	364	313	0	663	955	0	0	644	475	644	725	0	616	620	471	446	0	531	423	0
F2_20_2mm_count																				
R	87225	90059	88841	86986	80981	88451	89702	85432	87815	84804	81371	86645	83308	85064	83106	88706	89318	84759	83902	90373
T	2055	0	2448	2486	4156	2670	2254	0	2815	0	3693	3166	3564	0	4214	2410	2196	0	3267	1876
A	3773	1774	2099	1776	0	1651	1377	1950	2343	2577	0	2253	0	2537	0	2025	1487	2627	0	1434
C	0	1333	2548	0	5377	3164	2603	4157	0	4009	5671	3872	4543	4248	4740	0	2935	4142	4847	2253
G	2883	2770	0	4688	5422	0	0	4397	2963	4546	5201	0	4521	4087	3876	2795	0	4408	3920	0
F2_20_3mm_count																				
R	155975	165226	163673	159067	146930	165661	168137	155571	164016	154620	147334	161162	150708	154980	150366	166093	165501	153299	149470	166234
T	7741	0	7373	6989	10944	7178	6339	0	7590	0	10268	8306	10042	0	11439	6914	6766	0	10208	6668
A	12947	6005	6906	4936	0	4791	4103	5845	6373	7858	0	6543	0	7668	0	5569	4886	7506	0	5192
C	0	5608	8167	0	14070	8489	7540	12819	0	11829	15075	10108	12891	12379	13638	0	8966	12982	14462	8025
G	9456	9280	0	15127	14175	0	0	11884	8140	11812	13442	0	12478	11092	10676	7543	0	12332	11979	0
F2_20_4mm_count																				
R	179470	194028	193993	187085	171423	199623	203551	183904	197902	181729	172095	193173	175979	183099	175685	200319	199309	179514	172881	198502
T	15068	0	13028	11657	17591	11924	10667	0	12228	0	16279	13497	16412	0	18495	11484	11297	0	16930	11773
A	23024	11035	12230	8491	0	8324	7043	9698	10338	12914	0	10799	0	12426	0	9492	8442	12717	0	9332
C	0	12404	14703	0	22942	14083	12693	21111	0	20079	24372	16485	21797	20620	22212	0	14906	21797	23895	14347
G	16392	16487	0	26721	21998	0	0	19241	13486	19232	21208	0	19766	17809	17562	12659	0	19926	20248	0
F2_20_5mm_count																				
R	150346	166240	167414	159466	145063	176652	180175	158106	173708	156622	146771	169318	149462	157805	150239	176232	174976	153425	146786	173199
T	18958	0	15592	13684	19920	13740	12390	0	14147	0	18687	15592	18733	0	20694	13463	13344	0	19002	14007
A	26893	13115	14777	10061	0	9212	8025	11093	11959	14582	0	12166	0	13864	0	11095	9675	14438	0	11107
C	0	16433	17684	0	26247	15863	14877	24581	0	22756	26306	18391	25173	23615	24768	0	17472	25080	26707	17154
G	19270	19679	0	32256	24237	0	0	21687	15653	21507	23703	0	22099	20183	19766	14677	0	22524	22972	0
F2_20_6mm_count																				
R	96352	109082	110903	105193	94540	120146	123463	104712	117226	103589	96146	114558	98069	104593	98817	119521	118617	101145	96119	116727
T	17452	0	13981	11703	16891	11591	10449	0	12044	0	15505	13054	15592	0	17395	11720	11266	0	15868	12282
A	23265	11547	13069	8595	0	7863	6946	9542	10481	12385	0	10198	0	11631	0	9560	8172	12311	0	9756
C	0	15823	15584	0	22186	13937	12679	21020	0	19369	21994	15727	21166	19869	20650	0	15482	21396	22315	14772
G	16468	17085	0	28046	19920	0	0	18263	13786	18194	19892	0	18710	17444	16675	12736	0	18685	19235	0
F2_20_7mm_count																				
R	49401	56690	58637	54831	48793	65056	67133	55163	63161	54948	49713	61740	51158	55454	51764	64621	64523	53354	49836	62551
T	11735	0	9524	7871	11121	7826	6973	0	7923	0	10281	8641	10058	0	11310	7987	7350	0	10555	8260
A	15684	7852	9100	5747	0	5415	4702	6264	7089	8087	0	6627	0	7574	0	6503	5602	7912	0	6601
C	0	11358	10318	0	14719	9282	8771	14140	0	12685	14433	10571	14132	13070	13256	0	10104	14234	14603	10167
G	10759	11679	0	19130	12946	0	0	12012	9406	11859	13152	0	12231	11481	11249	8468	0	12079	12585	0
F2_20_8mm_count																				
R	19956	23554	24853	23299	20579	28576	29968	23511	27580	23329	21341	26897	21343	23613	21897	27992	28489	22771	21153	27207
T	6489	0	5252	4068	5835	4215	3525	0	4164	0	5170	4607	5323	0	5774	4330	3947	0	5538	4423
A	8444	4298	5045	2999	0	2782	2493	3242	3834	4171	0	3437	0	4024	0	3581	2828	4264	0	3547
C	0	6402	5509	0	7632	5086	4673	7515	0	6764	7486	5718	7440	6962	6945	0	5395	7445	7449	5482
G	5770	6405	0	10293	6613	0	0	6391	5081	6395	6662	0	6553	6060	6043	4756	0	6179	6519	0
F2_20_9mm	40659	40659	40659	40659	40659	40659	40659	40659	40659	40659	40659	40659	40659	40659	40659	40659	40659	40659	40659	40659
R	6452	7660	8346	7645	6885	9985	10622	7943	9460	7792	6862	9494	7134	8125	7169	9726	9998	7701	6871	9394
T	2812	0	2228	1712	2371	1719	1483	0	1719	0	2177	1824	2125	0	2448	1748	1587	0	2241	1904
A	3500	1812	2129	1210	0	1219	1008	1420	1618	1687	0	1388	0	1592	0	1456	1181	1662	0	1447
C	0	2825	2321	0	3109	2101	1911	3091	0	2835	3167	2318	3085	2787	2899	0	2258	3076	3105	2279
G	2260	2727	0	4457	2659	0	0	2570	2227	2710	2818	0	2680	2520	2508	2094	0	2585	2807	0
F2_20_10mm_count																				
R	1629	2031	2213	2070	1790	2785	3059	2163	2631	2149	1872	2657	1886	2168	1896	2673	2797	2016	1833	2512
T	896	0	781	558	793	568	471	0	558	0	746	562	651	0	748	602	538	0	717	667
A	1201	588	747	404	0	377	342	441	579	520	0	469	0	503	0	504	399	590	0	508
C	0	924	742	0	1033	753	611	1026	0	917	970	795	1046	958	1001	0	749	1029	1019	796
G	757	940	0	1451	867	0	0	853	715	897	895	0	900	854	838	704	0	848	914	0

R2 20	1	2	3	4	5	6	7	8	9	10	11	12	13	14	15	16	17	18	19	20
R2_20_0mm_count																				
R	2503	2503	2503	2503	2503	2503	2503	2503	2503	2503	2503	2503	2503	2503	2503	2503	2503	2503	2503	2503
T	0	0	0	0	0	0	0	0	0	0	0	0	0	0	0	0	0	0	0	0
A	0	0	0	0	0	0	0	0	0	0	0	0	0	0	0	0	0	0	0	0
C	0	0	0	0	0	0	0	0	0	0	0	0	0	0	0	0	0	0	0	0
G	0	0	0	0	0	0	0	0	0	0	0	0	0	0	0	0	0	0	0	0
R2_20_1mm_count																				
R	24741	25119	24902	24802	24111	24711	24969	24382	24849	24221	23940	24367	23950	24038	23741	24880	24664	24142	24225	25218
T	238	0	297	247	436	372	259	0	276	0	447	475	529	0	611	242	357	0	361	189
A	459	210	296	204	0	240	192	264	295	389	0	328	0	425	0	274	272	352	0	118
C	0	141	293	0	685	465	368	598	0	638	865	618	800	771	866	0	495	689	686	263
G	350	318	0	535	556	0	0	544	368	540	536	0	509	554	570	392	0	605	516	0
R2_20_2mm_count																				
R	76364	79098	78288	77013	73155	78332	79096	75299	78341	74840	72556	76456	73401	74496	72830	78648	77891	74045	73073	78660
T	1967	0	2044	1879	3204	2188	1977	0	2070	0	3003	2652	3118	0	3708	1870	2134	0	3086	2081
A	3572	1622	1981	1405	0	1498	1242	1769	1780	2356	0	2182	0	2478	0	1748	1602	2416	0	1492
C	0	1341	2236	0	4189	2531	2234	4029	0	3815	5024	3259	4336	4177	4658	0	2922	4225	4602	2316
G	2646	2488	0	4252	4001	0	0	3452	2358	3538	3966	0	3694	3398	3353	2283	0	3863	3788	0
R2_20_3mm_count																				
R	126154	134109	133883	130022	121013	135276	137506	127055	134647	125872	121048	131622	122716	126198	122378	136108	134362	124537	121052	134464
T	6643	0	5865	5365	8731	5808	4997	0	5794	0	8024	6619	8218	0	9244	5197	5720	0	8311	5850
A	10979	5187	5599	3825	0	3870	3353	4662	4894	6477	0	5457	0	6278	0	4454	4193	6422	0	4437
C	0	4928	6419	0	11338	6812	5910	10616	0	10045	12098	8068	11108	10405	11502	0	7491	10838	12180	7015
G	7990	7542	0	12554	10684	0	0	9433	6431	9372	10596	0	9724	8885	8642	6007	0	9969	10223	0
R2_20_4mm_count																				
R	139216	151773	152026	146587	134319	157108	159939	143738	155036	142196	134928	151272	137172	142715	137419	157117	155464	140010	134597	154520
T	12194	0	10015	9056	13587	9054	8202	0	9368	0	12741	10556	12831	0	14405	8962	8879	0	13260	9627
A	18251	8575	9623	6576	0	6146	5221	7303	8159	10082	0	8400	0	9705	0	7336	6683	10141	0	7349
C	0	9957	11283	0	17943	10639	9585	16813	0	15990	18714	12719	17466	16340	17624	0	11921	17399	18979	11451
G	13286	12642	0	20728	17098	0	0	15093	10384	14679	16564	0	15478	14187	13499	9532	0	15397	16111	0
R2_20_5mm_count																				
R	115974	128947	129892	124519	112500	137280	140362	122773	135004	121479	114020	131360	115937	122386	116607	137054	135675	119130	113681	134425
T	14940	0	12338	10477	15369	10426	9405	0	10760	0	14267	11869	14557	0	15909	10399	10324	0	14739	10921
A	21344	10184	11394	7606	0	7207	6205	8446	9287	11200	0	9639	0	10784	0	8591	7548	11312	0	8555
C	0	12825	13643	0	20364	12354	11295	19038	0	17875	20919	14399	19525	18319	19235	0	13720	19469	20984	13366
G	15009	15311	0	24665	19034	0	0	17010	12216	16713	18061	0	17248	15778	15516	11223	0	17356	17863	0
R2_20_6mm_count																				
R	74140	83827	85582	81143	73036	92460	95308	80384	90306	79829	74188	88252	75674	80228	76323	92162	91596	78424	73837	90341
T	13247	0	10830	8952	12677	8925	8067	0	9299	0	11888	10032	11968	0	13157	8972	8705	0	12420	9398
A	18303	8861	10052	6609	0	6108	5214	7031	8007	9613	0	7947	0	9223	0	7353	6280	9414	0	7319
C	0	12390	11896	0	17172	10867	9771	16697	0	15084	17046	12129	16446	15355	16082	0	11779	16252	17205	11302
G	12670	13282	0	21656	15475	0	0	14248	10748	13834	15238	0	14272	13554	12798	9873	0	14270	14898	0
R2_20_7mm_count																				
R	37442	43153	44528	42038	37360	49975	51548	42189	48227	41796	38518	47070	38777	42426	39455	49297	49163	40667	37924	48043
T	9056	0	7261	5968	8472	5910	5279	0	6115	0	7711	6701	7880	0	8594	6183	5674	0	7968	6323
A	12143	5752	6860	4338	0	3915	3478	4698	5375	6169	0	5162	0	5817	0	4859	4171	6088	0	4915
C	0	8901	8243	0	11020	7092	6587	10862	0	9815	10952	7959	10871	9938	10407	0	7884	10904	11156	7611
G	8251	9086	0	14548	10040	0	0	9143	7175	9112	9711	0	9364	8711	8436	6553	0	9233	9844	0
R2_20_8mm_count																				
R	15120	17641	18901	17275	15365	21449	22476	17605	20552	17505	15936	20249	16061	17696	16403	21219	21267	16861	15660	20579
T	4828	0	3783	3147	4316	3135	2702	0	3153	0	4005	3479	4030	0	4378	3125	2993	0	4116	3279
A	6383	3091	3581	2352	0	2114	1850	2490	2863	3154	0	2597	0	3009	0	2641	2132	3139	0	2534
C	0	4998	4220	0	5796	3787	3457	5555	0	5098	5591	4160	5562	5184	5238	0	4093	5743	5631	4093
G	4154	4755	0	7711	5008	0	0	4835	3917	4728	4953	0	4832	4596	4466	3500	0	4742	5078	0
R2_20_9mm_count																				
R	4772	5678	6219	5692	4992	7477	7805	5850	7126	5832	5334	6998	5245	6023	5378	7250	7397	5738	5055	6965
T	2065	0	1700	1335	1805	1253	1097	0	1269	0	1581	1403	1629	0	1798	1351	1254	0	1680	1367
A	2641	1242	1566	942	0	853	789	1019	1134	1288	0	1033	0	1241	0	1039	862	1286	0	1097
C	0	2184	1681	0	2310	1583	1475	2346	0	2103	2233	1732	2312	2063	2144	0	1653	2279	2334	1737
G	1688	2062	0	3197	2059	0	0	1951	1637	1943	2018	0	1980	1839	1846	1526	0	1863	2097	0
R2_20_10mm_count																				
R	1167	1475	1687	1529	1331	2073	2209	1624	1924	1565	1387	1958	1335	1570	1391	2010	2067	1448	1376	1874
T	710	0	555	404	565	416	373	0	373	0	544	452	535	0	615	410	400	0	523	470
A	833	435	489	293	0	271	250	346	438	416	0	310	0	383	0	369	288	412	0	369
C	0	726	569	0	719	540	468	759	0	680	693	580	762	728	693	0	545	776	753	587
G	590	664	0	1074	685	0	0	571	565	639	676	0	668	619	601	511	0	664	648	0

No_LNA_21	1	2	3	4	5	6	7	8	9	10	11	12	13	14	15	16	17	18	19	20	21
No_LNA_21_0mm_count																					
R	5190	5190	5190	5190	5190	5190	5190	5190	5190	5190	5190	5190	5190	5190	5190	5190	5190	5190	5190	5190	5190
T	0	0	0	0	0	0	0	0	0	0	0	0	0	0	0	0	0	0	0	0	0
A	0	0	0	0	0	0	0	0	0	0	0	0	0	0	0	0	0	0	0	0	0
C	0	0	0	0	0	0	0	0	0	0	0	0	0	0	0	0	0	0	0	0	0
G	0	0	0	0	0	0	0	0	0	0	0	0	0	0	0	0	0	0	0	0	0
1 mm																					
R	26610	26048	26559	26801	25857	26696	26815	26385	26832	26132	25925	26520	25977	26159	25924	26818	26571	26038	25814	26573	26605
T	347	0	410	324	524	347	280	0	308	0	463	379	500	0	558	351	385	0	485	353	421
A	310	380	304	229	0	252	247	330	252	376	0	300	0	364	0	223	293	400	0	286	336
C	0	598	407	0	645	385	338	492	0	570	720	481	647	606	697	0	431	632	797	468	318
G	413	654	0	326	654	0	0	473	288	602	572	0	556	551	501	288	0	610	584	0	0
2 mm																					
R	66331	63786	65654	66651	62151	66209	66696	64580	66818	63960	62168	65380	62740	63910	62593	67028	65633	63028	61928	65702	66149
T	1769	0	2206	1619	2683	1927	1648	0	1713	0	2426	2062	2511	0	2914	1565	1944	0	2650	1982	2117
A	1488	1786	1632	1378	0	1372	1415	1692	1349	1915	0	1643	0	1790	0	1324	1572	1970	0	1532	1581
C	0	2831	2024	0	3369	2008	1757	2699	0	2956	3838	2431	3346	3210	3425	0	2367	3412	3808	2300	1669
G	1928	3113	0	1868	3313	0	0	2545	1636	2685	3084	0	2919	2606	2584	1599	0	3106	3130	0	0
3mm																					
R	108883	102949	106823	109713	98642	108558	108841	104496	109511	102717	98518	106229	100077	102789	99936	110158	106691	101418	98345	107458	108185
T	4646	0	5657	4312	6709	4791	4571	0	4557	0	6266	5492	6352	0	7165	4388	5346	0	6707	5090	5528
A	3845	4622	4201	3560	0	3515	3913	4436	3796	4965	0	4213	0	4806	0	3453	4219	4929	0	3932	4008
C	0	7199	5549	0	8700	5366	4905	6996	0	7473	9795	6296	8425	8037	8772	0	5974	8404	9549	5750	4509
G	4856	7460	0	4645	8179	0	0	6302	4366	7075	7651	0	7376	6598	6357	4231	0	7479	7629	0	0
4mm																					
R	126333	117364	123196	127530	110209	125776	126029	119531	127303	117088	111129	122703	113096	117319	112715	128404	123072	114986	110414	123990	125658
T	7583	0	9299	7256	10931	8134	7817	0	7367	0	10090	8827	10132	0	11494	7309	8586	0	10550	8413	8961
A	6384	7328	7044	6106	0	5731	6538	7135	6295	7698	0	6854	0	7602	0	5738	6950	7998	0	6412	6675
C	0	11638	8838	0	14199	8736	7993	11580	0	12277	15080	9993	13488	12791	13677	0	9769	13624	15121	9562	7083
G	8077	12047	0	7485	13038	0	0	10131	7412	11314	12078	0	11661	10665	10491	6926	0	11769	12292	0	0
5mm																					
R	110360	100863	107244	112126	92953	109151	109968	102727	111094	100325	93799	106512	95578	100480	95936	112164	106546	98110	93710	107840	109268
T	8948	0	10579	8418	12701	9501	8954	0	8686	0	11302	10075	11639	0	12628	8625	10076	0	11623	9719	10738
A	7323	8236	7962	6570	0	6796	7410	8278	7416	8841	0	7690	0	8447	0	6716	8184	9028	0	7164	7629
C	0	13370	10149	0	15996	10486	9602	13364	0	13971	16978	11657	15573	14652	15432	0	11128	15708	16744	11211	8299
G	9303	13465	0	8820	14284	0	0	11565	8738	12797	13855	0	13144	12355	11938	8429	0	13088	13857	0	0
6mm																					
R	74473	66841	72285	76067	60483	73576	73906	68159	75142	66130	61420	71461	62800	66564	63150	75937	71479	65059	61464	72576	73481
T	7612	0	8921	7396	10642	8350	7741	0	7460	0	9694	8908	9685	0	10553	7571	8521	0	10038	8156	9229
A	6538	7069	6751	5721	0	5865	6519	7199	6476	7560	0	6445	0	7236	0	5998	6897	7597	0	6468	6726
C	0	11538	8757	0	13655	8923	8548	11608	0	12005	13946	9900	13199	12466	12656	0	9817	13230	13718	9514	7278
G	8091	11266	0	7530	11934	0	0	9748	7636	11019	11654	0	11030	10448	10355	7208	0	10828	11494	0	0
7mm																					
R	39696	34809	38420	40763	31324	39123	39375	35858	40017	34909	31679	38066	32682	34832	32930	40828	38192	33912	31668	38647	38894
T	5233	0	5912	4945	6814	5617	5160	0	5011	0	6390	5732	6215	0	6813	5170	5654	0	6542	5397	6203
A	4267	4759	4559	3844	0	3846	4348	4795	4402	4799	0	4119	0	4658	0	3844	4573	4910	0	4157	4436
C	0	7696	5769	0	8814	6074	5777	7539	0	7802	8972	6743	8679	8236	8127	0	6241	8695	8990	6459	5127
G	5464	7396	0	5108	7708	0	0	6468	5230	7150	7619	0	7084	6934	6790	4818	0	7143	7460	0	0
8mm																					
R	16694	14527	16302	17562	12688	16391	16570	14951	16924	14434	12905	16271	13152	14575	13559	17343	16157	13914	12875	16161	16142
T	2749	0	3148	2542	3415	2982	2653	0	2520	0	3246	2833	3125	0	3393	2608	2792	0	3269	2907	3253
A	2199	2371	2280	1970	0	2044	2270	2447	2309	2505	0	2030	0	2334	0	2088	2322	2586	0	2167	2509
C	0	3907	2828	0	4546	3141	3065	3930	0	4021	4500	3424	4692	4076	4126	0	3287	4500	4653	3323	2654
G	2916	3753	0	2484	3909	0	0	3230	2805	3598	3907	0	3589	3573	3480	2519	0	3558	3761	0	0
9 mm																					
R	5692	4886	5608	6054	4243	5707	5631	5034	5852	4891	4270	5659	4465	4915	4445	6024	5569	4811	4282	5487	5441
T	1224	0	1252	1063	1443	1191	1105	0	1063	0	1228	1151	1246	0	1456	1110	1163	0	1306	1185	1414
A	904	979	1007	795	0	814	962	1005	937	979	0	824	0	962	0	808	957	1001	0	915	1031
C	0	1621	1177	0	1840	1332	1346	1641	0	1660	1906	1410	1874	1643	1695	0	1355	1773	1879	1457	1158
G	1224	1558	0	1132	1518	0	0	1364	1192	1514	1640	0	1459	1524	1448	1102	0	1459	1577	0	0
10mm																					
R	1487	1159	1513	1633	1060	1504	1519	1277	1531	1240	1119	1497	1102	1286	1114	1588	1474	1187	1108	1401	1381
T	355	0	362	322	427	368	336	0	340	0	392	372	399	0	429	341	365	0	421	398	450
A	312	331	290	244	0	242	268	325	285	310	0	243	0	260	0	260	278	333	0	269	315
C	0	542	378	0	602	429	420	525	0	502	537	431	559	539	520	0	426	562	564	475	397
G	389	511	0	344	454	0	0	416	387	491	495	0	483	458	480	354	0	461	450	0	0

F2 24A	1	2	3	4	5	6	7	8	9	10	11	12	13	14	15	16	17	18	19	20	21
F2_24A_0mm_count																					
R	531	531	531	531	531	531	531	531	531	531	531	531	531	531	531	531	531	531	531	531	531
T	0	0	0	0	0	0	0	0	0	0	0	0	0	0	0	0	0	0	0	0	0
A	0	0	0	0	0	0	0	0	0	0	0	0	0	0	0	0	0	0	0	0	0
C	0	0	0	0	0	0	0	0	0	0	0	0	0	0	0	0	0	0	0	0	0
G	0	0	0	0	0	0	0	0	0	0	0	0	0	0	0	0	0	0	0	0	0
1mm																					
R	2709	2641	2682	2681	2511	2636	2675	2612	2660	2611	2570	2657	2593	2605	2607	2684	2680	2581	2555	2671	2705
T	15	0	23	22	61	41	41	0	34	0	41	41	36	0	59	21	40	0	57	38	20
A	21	22	24	20	0	19	15	20	25	27	0	21	0	29	0	28	19	32	0	12	21
C	0	55	37	0	96	70	35	52	0	46	93	47	66	57	54	0	27	80	94	45	20
G	21	48	0	43	98	0	0	82	47	82	62	0	71	75	46	33	0	73	60	0	0
2 mm																					
R	15502	15021	15032	13459	11982	14264	14785	14224	13863	14321	13501	14457	14353	14455	14400	14732	14897	14259	14091	14648	15409
T	103	0	327	686	1005	678	436	0	530	0	553	504	407	0	546	322	433	0	479	454	185
A	115	170	187	629	0	188	230	408	588	288	0	221	0	247	0	289	167	296	0	199	138
C	0	283	328	0	1295	744	423	412	0	450	942	692	515	402	475	0	377	501	685	573	142
G	154	400	0	1100	1592	0	0	830	893	815	878	0	599	770	453	531	0	818	619	0	0
3mm																					
R	83665	79354	77553	73415	62615	74010	77019	74869	73593	75113	70030	75536	74117	75699	73504	77523	77945	74936	71003	75972	83434
T	1345	0	3948	4818	7104	5525	4361	0	4599	0	4556	4478	4076	0	5074	3313	4163	0	4623	4327	1731
A	1233	1949	2403	4052	0	2243	2310	3442	4136	2728	0	2507	0	2721	0	2872	1917	3004	0	2193	1249
C	0	3044	3903	0	9050	6029	4117	3673	0	3946	6950	5286	4508	3930	4615	0	3782	4057	6434	5315	1393
G	1564	3460	0	5522	9038	0	0	5823	5479	6020	6271	0	5106	5457	4614	4099	0	5810	5747	0	0
4mm																					
R	173352	160685	162008	163654	137594	160853	163218	155403	162043	154515	143910	159038	149446	155144	148394	165513	161678	153056	144818	160590	170968
T	6920	0	12053	10637	16104	12337	11226	0	11023	0	13300	12290	13109	0	15151	10259	12170	0	13540	11954	9332
A	6383	7999	8369	8673	0	7642	8388	10361	9574	9525	0	8762	0	9591	0	8203	7902	9723	0	7915	6623
C	0	12491	11947	0	21339	13545	11545	13894	0	14686	20052	14287	16391	15061	16703	0	12627	15581	19452	13918	7454
G	7722	13202	0	11413	19340	0	0	14719	11737	15651	17115	0	15431	14581	14129	10402	0	16017	16567	0	0
5mm																					
R	179925	163534	171512	178081	146158	173156	174773	163391	176130	160188	149090	169158	153013	161151	153432	178416	170284	157874	149240	171594	176813
T	12834	0	16906	13794	20277	15877	14803	0	14062	0	18446	16687	18343	0	20319	14165	16335	0	18950	15837	16157
A	10817	12450	12566	10836	0	10992	11656	13711	12374	13874	0	12328	0	13492	0	10887	12552	14239	0	11266	11284
C	0	20738	16161	0	27113	17120	15913	21102	0	22030	26924	18972	24565	22621	23981	0	17974	23898	26428	18448	12891
G	13569	20423	0	14434	23597	0	0	18941	14579	21053	22685	0	21224	19881	19413	13677	0	21134	22527	0	0
6mm																					
R	128756	115392	123966	129710	103275	125695	126692	116813	128567	113752	104956	122425	107344	113805	107908	129997	122799	111750	104638	124034	126031
T	12758	0	15257	12694	18067	14389	13511	0	12557	0	16628	14949	16561	0	18283	13042	14793	0	17186	14107	15638
A	10490	11908	11670	10042	0	9916	10896	12409	11430	12603	0	10983	0	12093	0	10142	11525	12825	0	10719	11429
C	0	19290	14806	0	23789	15699	14600	19508	0	20224	23995	17342	22699	21445	21938	0	16582	22310	24088	16839	12601
G	13695	19109	0	13253	20568	0	0	16969	13145	19120	20120	0	19095	18356	17570	12518	0	18814	19787	0	0
7mm																					
R	71024	62163	68391	72816	54818	69568	70085	63316	71215	61483	56283	67663	57670	61624	58429	72220	67505	60175	56086	68157	68937
T	8828	0	10310	8804	12479	9899	9145	0	8670	0	11090	10204	11002	0	12069	8881	10053	0	11440	9664	11105
A	7522	8237	8103	6603	0	6695	7542	8557	7727	8577	0	7270	0	8258	0	7082	8026	8570	0	7394	8032
C	0	13324	10146	0	15996	10788	10178	13454	0	13999	15975	11813	15358	14451	14504	0	11366	15693	16125	11735	8876
G	9576	13226	0	8727	13657	0	0	11623	9338	12891	13602	0	12920	12617	11948	8767	0	12512	13299	0	0
8mm																					
R	31070	26398	29868	32421	23155	30465	30674	27404	31353	26407	23901	29879	24389	26328	24738	31956	29777	25440	23627	29655	29842
T	4791	0	5540	4506	6365	5268	4891	0	4610	0	5803	5236	5637	0	6358	4805	5152	0	6027	5269	6060
A	4071	4498	4372	3394	0	3611	4053	4508	4136	4490	0	3743	0	4254	0	3754	4177	4556	0	3946	4375
C	0	7140	5389	0	8491	5825	5551	7163	0	7391	8210	6311	8381	7769	7618	0	6063	8462	8538	6299	4892
G	5237	7133	0	4848	7158	0	0	6094	5070	6881	7255	0	6762	6818	6455	4654	0	6711	6977	0	0
9mm																					
R	10951	9221	10812	11625	8026	10814	10892	9587	11180	9269	8154	10669	8283	9155	8611	11408	10466	8906	8091	10389	10479
T	2161	0	2247	1998	2633	2337	2054	0	1909	0	2448	2216	2401	0	2668	2096	2272	0	2568	2296	2572
A	1743	1924	1816	1459	0	1520	1779	1952	1847	1899	0	1536	0	1781	0	1601	1808	1867	0	1689	1942
C	0	3154	2305	0	3589	2509	2455	3118	0	3071	3507	2759	3656	3337	3132	0	2634	3613	3538	2806	2187
G	2325	2881	0	2098	2932	0	0	2523	2244	2941	3071	0	2840	2907	2769	2075	0	2794	2983	0	0
10mm																					
R	3255	2561	3071	3507	2284	3134	3166	2763	3335	2681	2270	3166	2360	2549	2413	3310	3061	2563	2265	2985	3025
T	721	0	832	712	910	815	703	0	670	0	859	744	768	0	914	762	783	0	878	795	923
A	584	667	660	479	0	528	634	660	613	654	0	479	0	608	0	578	608	686	0	633	660
C	0	1140	833	0	1216	919	893	1075	0	1087	1251	1007	1232	1202	1091	0	944	1193	1234	983	788
G	836	1028	0	698	986	0	0	898	778	974	1016	0	1036	1037	978	746	0	954	1019	0	0

24D_R2	1	2	3	4	5	6	7	8	9	10	11	12	13	14	15	16	17	18	19	20	21
24D_R2_0mm_count																					
R	531	531	531	531	531	531	531	531	531	531	531	531	531	531	531	531	531	531	531	531	531
T	0	0	0	0	0	0	0	0	0	0	0	0	0	0	0	0	0	0	0	0	0
A	0	0	0	0	0	0	0	0	0	0	0	0	0	0	0	0	0	0	0	0	0
C	0	0	0	0	0	0	0	0	0	0	0	0	0	0	0	0	0	0	0	0	0
G	0	0	0	0	0	0	0	0	0	0	0	0	0	0	0	0	0	0	0	0	0
1mm																					
R	5485	5382	5448	5507	5339	5398	5399	5371	5460	5381	5235	5293	5303	5273	5250	5453	5183	5086	4957	4992	5456
T	26	0	58	14	61	75	49	0	45	0	71	82	59	0	96	37	105	0	110	187	41
A	27	41	32	22	0	28	52	41	38	44	0	54	0	54	0	44	62	100	0	91	33
C	0	67	44	0	102	81	82	83	0	65	183	153	157	144	150	0	232	245	415	312	52
G	44	92	0	39	80	0	0	87	39	92	93	0	63	111	86	48	0	151	100	0	0
2mm																					
R	39383	38489	37876	38670	36307	36776	36837	37358	38001	36320	35614	35442	35619	36002	35024	38087	34900	34251	33096	33717	38813
T	281	0	903	386	1020	1213	1174	0	599	0	1010	1473	1216	0	1562	523	1748	0	1538	2191	570
A	284	380	647	551	0	809	858	740	716	855	0	1118	0	978	0	847	1221	1376	0	1641	419
C	0	709	916	0	1842	1544	1473	1330	0	1645	2441	2309	2327	2069	2515	0	2473	2686	3981	2793	540
G	394	764	0	735	1173	0	0	914	1026	1522	1277	0	1180	1293	1241	885	0	2029	1727	0	0
3mm																					
R	115595	109619	110375	113206	102453	108824	109011	107371	111528	104372	100492	105492	101564	103828	100364	112020	105239	100121	97028	104590	111997
T	2826	0	5086	3332	5901	5351	5077	0	4017	0	5913	6030	6324	0	7416	3917	6263	0	7226	6529	4767
A	2510	3403	3773	3200	0	3943	4231	4248	3925	4676	0	4774	0	4914	0	3769	4986	5894	0	4868	3599
C	0	5417	4910	0	8662	6026	5825	7081	0	7914	10742	7848	9638	8840	10088	0	7656	9971	11932	8157	3781
G	3213	5705	0	4406	7128	0	0	5444	4674	7182	6997	0	6618	6562	6276	4438	0	8158	7958	0	0
4mm																					
R	164727	152820	158335	163803	141600	158968	159499	152594	161650	148602	140767	153974	143261	148223	142143	163086	153817	144523	138395	155294	160230
T	7971	0	11089	8279	13331	10380	9974	0	9088	0	12442	11675	12869	0	14771	8816	11822	0	14097	11254	11153
A	6938	8259	8257	7027	0	7492	8197	9023	8180	9792	0	9029	0	9751	0	7603	9510	10483	0	8612	8391
C	0	13554	10844	0	18135	11685	10855	14542	0	15609	20013	13847	18137	16985	18381	0	13376	18294	20386	13365	8751
G	8889	13892	0	9416	15459	0	0	12366	9607	14522	15303	0	14258	13566	13230	9020	0	15225	15647	0	0
5mm																					
R	154017	140611	149587	155994	129175	151495	151865	142208	154179	138317	129307	146424	132431	138442	131802	155382	146594	135150	128279	148233	151317
T	11643	0	13913	11092	16638	13071	12393	0	11626	0	15969	14231	15770	0	17759	11843	14240	0	17014	13356	14388
A	9760	11051	10880	8946	0	9173	10145	11430	10152	12109	0	10734	0	11830	0	9357	11259	12510	0	10447	10734
C	0	18086	13648	0	22719	14289	13625	18633	0	19514	23850	16639	21946	20708	21846	0	15935	21963	23777	15992	11589
G	12608	18280	0	11996	19496	0	0	15757	12071	18088	18902	0	17881	17048	16621	11446	0	18405	18958	0	0
6mm																					
R	108160	96979	105054	110738	87503	106761	107163	98726	109037	96057	88138	103379	90615	95864	91046	110222	103484	93789	88121	104644	106295
T	11039	0	12774	10372	15037	11904	11271	0	10618	0	14206	12675	13676	0	15533	10748	12320	0	14458	11884	13467
A	9007	10244	9849	8093	0	8253	9139	10270	9156	10805	0	9119	0	10706	0	8519	10034	10918	0	9114	9757
C	0	16618	12275	0	20433	13034	12379	16620	0	17237	20730	14779	19533	17921	18597	0	14114	19403	20462	14310	10433
G	11746	16111	0	10749	16979	0	0	14336	11141	15853	16878	0	16128	15461	14776	10463	0	15842	16911	0	0
7mm																					
R	59528	52182	57761	61665	46208	58904	58778	53565	60147	52123	47409	56680	48433	51900	48731	61109	56435	50661	46817	57165	58181
T	7490	0	8814	7113	10251	8212	7806	0	7252	0	9345	8609	9136	0	10498	7551	8617	0	9626	8396	9091
A	6317	6950	6639	5450	0	5579	6453	7015	6323	7235	0	6193	0	6867	0	5787	6711	7311	0	6268	6704
C	0	11260	8380	0	13686	8899	8557	11297	0	11724	13692	10112	13215	12247	12273	0	9831	13009	13751	9765	7618
G	8259	11202	0	7366	11449	0	0	9717	7872	10512	11148	0	10810	10580	10092	7147	0	10613	11400	0	0
8mm																					
R	26524	22813	25794	27929	20022	26261	26504	23660	27087	22802	20570	25553	20947	22968	21236	27457	25440	21929	20376	25495	25806
T	4189	0	4851	4041	5530	4700	4109	0	3898	0	5061	4623	4941	0	5581	4127	4597	0	5181	4567	5125
A	3578	3786	3716	2958	0	3009	3479	3862	3618	3889	0	3192	0	3654	0	3170	3623	3920	0	3379	3787
C	0	6231	4542	0	7329	4933	4811	6174	0	6251	7280	5535	7321	6625	6612	0	5243	7321	7315	5462	4185
G	4612	6073	0	3975	6022	0	0	5207	4300	5961	5992	0	5694	5656	5474	4149	0	5733	6031	0	0
9mm																					
R	9565	8172	9412	10342	6917	9471	9738	8379	10073	8153	7269	9375	7424	8297	7471	10024	9267	7832	7237	9079	9126
T	1887	0	2001	1781	2395	2032	1788	0	1617	0	2157	1946	2104	0	2388	1888	1991	0	2190	2023	2279
A	1585	1594	1684	1198	0	1399	1504	1671	1518	1704	0	1400	0	1543	0	1452	1562	1762	0	1534	1703
C	0	2737	2060	0	3192	2255	2127	2802	0	2763	3134	2436	3103	2866	2814	0	2337	3087	3146	2521	2049
G	2120	2654	0	1836	2653	0	0	2305	1949	2537	2597	0	2526	2451	2484	1793	0	2476	2584	0	0
10mm																					
R	2781	2291	2720	3012	1965	2749	2845	2369	2885	2364	1967	2720	1983	2305	2128	2864	2661	2188	1997	2607	2595
T	632	0	710	629	775	707	584	0	565	0	724	664	732	0	812	685	748	0	741	716	821
A	531	539	566	407	0	482	512	572	551	499	0	452	0	522	0	497	495	576	0	510	594
C	0	968	701	0	1104	759	756	965	0	972	1080	861	1122	1027	904	0	793	1100	1075	864	687
G	753	899	0	649	853	0	0	791	696	862	926	0	860	843	853	651	0	833	884	0	0

No_LNA_18_	1	2	3	4	5	6	7	8	9	10	11	12	13	14	15	16	17	18			
R	92.55	95.68	96.13	94.74	92.17	96.07	97.14	93.63	96.26	93.64	92.36	95.15	92.93	93.87	92.66	96.17	95.86	92.99			
T	1.73	0.00	1.22	1.45	2.21	1.34	0.92	0.00	1.33	0.00	1.97	1.71	1.89	0.00	2.30	1.36	1.22	0.00			
A	3.39	1.51	1.22	0.74	0.00	0.98	0.75	1.20	1.08	1.40	0.00	1.24	0.00	1.40	0.00	1.14	1.05	1.57			
C	0.00	0.98	1.42	0.00	2.75	1.61	1.20	2.76	0.00	2.54	3.14	1.90	2.66	2.62	2.88	0.00	1.88	2.67			
G	2.34	1.82	0.00	3.07	2.86	0.00	0.00	2.41	1.33	2.42	2.54	0.00	2.53	2.12	2.16	1.33	0.00	2.77			
F2_18_1mm_count																					
R	96.08	97.88	96.36	94.11	89.80	95.26	96.59	93.90	94.37	93.86	90.09	94.09	92.52	93.34	91.86	95.24	98.18	96.48			
T	0.80	0.00	1.23	1.70	2.73	1.73	1.38	0.00	1.93	0.00	2.55	2.18	2.06	0.00	2.88	1.57	0.56	0.00			
A	1.85	0.64	1.09	1.25	0.00	0.81	0.61	1.05	1.55	1.29	0.00	1.17	0.00	1.63	0.00	1.35	0.43	0.73			
C	0.00	0.45	1.32	0.00	3.72	2.19	1.42	2.17	0.00	1.84	3.85	2.57	2.40	2.13	2.80	0.00	0.83	1.32			
G	1.27	1.03	0.00	2.95	3.76	0.00	0.00	2.88	2.15	3.02	3.51	0.00	3.02	2.90	2.46	1.85	0.00	1.46			
R2_18_1mm_count																					
R	95.87	97.09	96.26	96.12	92.77	95.27	96.19	94.05	95.93	93.03	91.47	94.03	91.69	92.30	90.94	96.05	96.04	94.89			
T	1.00	0.00	1.28	1.08	1.95	1.61	1.22	0.00	1.29	0.00	2.23	2.05	2.34	0.00	2.86	1.32	1.15	0.00			
A	1.73	0.97	1.27	0.74	0.00	1.08	0.80	1.19	1.21	1.75	0.00	1.48	0.00	1.82	0.00	1.06	0.98	1.13			
C	0.00	0.59	1.19	0.00	2.91	2.04	1.78	2.62	0.00	2.71	3.68	2.44	3.37	3.27	3.64	0.00	1.83	2.29			
G	1.39	1.35	0.00	2.07	2.37	0.00	0.00	2.14	1.57	2.51	2.62	0.00	2.60	2.61	2.55	1.56	0.00	1.69			
No_LNA_20_	1	2	3	4	5	6	7	8	9	10	11	12	13	14	15	16	17	18	19	20	
R	92.99	95.75	96.65	95.45	92.97	96.75	97.53	94.35	96.53	94.27	93.45	95.63	93.85	94.64	93.64	96.50	96.43	93.65	92.70	96.26	
T	1.68	0.00	1.07	1.31	2.04	1.15	0.81	0.00	1.27	0.00	1.68	1.55	1.61	0.00	2.02	1.17	0.97	0.00	2.12	1.22	
A	3.09	1.47	1.06	0.63	0.00	0.79	0.70	1.13	0.97	1.28	0.00	1.08	0.00	1.15	0.00	1.13	0.95	1.51	0.00	0.87	
C	0.00	0.95	1.22	0.00	2.53	1.31	0.97	2.36	0.00	2.27	2.59	1.74	2.33	2.31	2.50	0.00	1.65	2.34	2.95	1.65	
G	2.25	1.82	0.00	2.61	2.46	0.00	0.00	2.17	1.23	2.18	2.28	0.00	2.21	1.90	1.85	1.20	0.00	2.50	2.22	0.00	
F2_20_1mm_count																					
R	95.42	97.57	97.07	94.68	89.78	95.55	97.20	94.79	94.91	94.83	92.03	94.79	93.61	94.15	93.28	95.37	97.01	94.95	95.01	98.00	
T	0.98	0.00	1.00	1.59	2.82	1.79	1.12	0.00	1.67	0.00	2.05	1.83	1.67	0.00	2.44	1.54	1.07	0.00	1.39	0.63	
A	2.11	0.75	0.85	1.00	0.00	0.72	0.44	0.97	1.47	1.07	0.00	1.02	0.00	1.37	0.00	1.26	0.59	0.96	0.00	0.58	
C	0.00	0.39	1.09	0.00	3.49	1.94	1.24	1.60	0.00	1.46	2.94	2.37	2.19	1.94	2.34	0.00	1.34	1.91	1.86	0.80	
G	1.49	1.28	0.00	2.72	3.92	0.00	0.00	2.64	1.95	2.64	2.97	0.00	2.53	2.54	1.93	1.83	0.00	2.18	1.73	0.00	
R2_20_1mm_count																					
R	95.94	97.41	96.56	96.18	93.50	95.82	96.82	94.55	96.36	93.92	92.83	94.49	92.87	93.21	92.06	96.48	95.64	93.62	93.94	97.79	
T	0.92	0.00	1.15	0.96	1.69	1.44	1.00	0.00	1.07	0.00	1.73	1.84	2.05	0.00	2.37	0.94	1.38	0.00	1.40	0.73	
A	1.78	0.81	1.15	0.79	0.00	0.93	0.74	1.02	1.14	1.51	0.00	1.27	0.00	1.65	0.00	1.06	1.05	1.36	0.00	0.46	
C	0.00	0.55	1.14	0.00	2.66	1.80	1.43	2.32	0.00	2.47	3.35	2.40	3.10	2.99	3.36	0.00	1.92	2.67	2.66	1.02	
G	1.36	1.23	0.00	2.07	2.16	0.00	0.00	2.11	1.43	2.09	2.08	0.00	1.97	2.15	2.21	1.52	0.00	2.35	2.00	0.00	
No LNA 1 mr	1	2	3	4	5	6	7	8	9	10	11	12	13	14	15	16	17	18	19	20	21
R	96.13	94.10	95.95	96.82	93.41	96.45	96.88	95.32	96.94	94.41	93.66	95.81	93.85	94.51	93.66	96.89	95.99	94.07	93.26	96.00	96.12
T	1.25	0.00	1.48	1.17	1.89	1.25	1.01	0.00	1.11	0.00	1.67	1.37	1.81	0.00	2.02	1.27	1.39	0.00	1.75	1.28	1.52
A	1.12	1.37	1.10	0.83	0.00	0.91	0.89	1.19	0.91	1.36	0.00	1.08	0.00	1.32	0.00	0.81	1.06	1.45	0.00	1.03	1.21
C	0.00	2.16	1.47	0.00	2.33	1.39	1.22	1.78	0.00	2.06	2.60	1.74	2.34	2.19	2.52	0.00	1.56	2.28	2.88	1.69	1.15
G	1.49	2.36	0.00	1.18	2.36	0.00	0.00	1.71	1.04	2.17	2.07	0.00	2.01	1.99	1.81	1.04	0.00	2.20	2.11	0.00	0.00
24D_F2 1mm																					
R	97.94	95.48	96.96	96.93	90.78	95.30	96.71	94.43	96.17	94.40	92.91	96.06	93.75	94.18	94.25	97.04	96.89	93.31	92.37	96.57	97.79
T	0.54	0.00	0.83	0.80	2.21	1.48	1.48	0.00	1.23	0.00	1.48	1.48	1.30	0.00	2.13	0.76	1.45	0.00	2.06	1.37	0.72
A	0.76	0.80	0.87	0.72	0.00	0.69	0.54	0.72	0.90	0.98	0.00	0.76	0.00	1.05	0.00	1.01	0.69	1.16	0.00	0.43	0.76
C	0.00	1.99	1.34	0.00	3.47	2.53	1.27	1.88	0.00	1.66	3.36	1.70	2.39	2.06	1.95	0.00	0.98	2.89	3.40	1.63	0.72
G	0.76	1.74	0.00	1.55	3.54	0.00	0.00	2.96	1.70	2.96	2.24	0.00	2.57	2.71	1.66	1.19	0.00	2.64	2.17	0.00	0.00
24D_R2 1 mm																					
R	98.26	96.42	97.60	98.66	95.65	96.70	96.72	96.22	97.81	96.40	93.78	94.82	95.00	94.46	94.05	97.69	92.85	91.11	88.80	89.43	97.74
T	0.47	0.00	1.04	0.25	1.09	1.34	0.88	0.00	0.81	0.00	1.27	1.47	1.06	0.00	1.72	0.66	1.88	0.00	1.97	3.35	0.73
A	0.48	0.73	0.57	0.39	0.00	0.50	0.93	0.73	0.68	0.79	0.00	0.97	0.00	0.97	0.00	0.79	1.11	1.79	0.00	1.63	0.59
C	0.00	1.20	0.79	0.00	1.83	1.45	1.47	1.49	0.00	1.16	3.28	2.74	2.81	2.58	2.69	0.00	4.16	4.39	7.43	5.59	0.93
G	0.79	1.65	0.00	0.70	1.43	0.00	0.00	1.56	0.70	1.65	1.67	0.00	1.13	1.99	1.54	0.86	0.00	2.71	1.79	0.00	0.00
No LNA 2 mr	1	2	3	4	5	6	7	8	9	10	11	12	13	14	15	16	17	18	19	20	21
R	92.75	89.19	91.80	93.20	86.91	92.58	93.26	90.30	93.43	89.43	86.93	91.42	87.73	89.36	87.52	93.72	91.77	88.13	86.59	91.87	92.50
T	2.47	0.00	3.08	2.26	3.75	2.69	2.30	0.00	2.40	0.00	3.39	2.88	3.51	0.00	4.07	2.19	2.72	0.00	3.71	2.77	2.96
A	2.08	2.50	2.28	1.93	0.00	1.92	1.98	2.37	1.89	2.68	0.00	2.30	0.00	2.50	0.00	1.85	2.20	2.75	0.00	2.14	2.21
C	0.00	3.96	2.83	0.00	4.71	2.81	2.46	3.77	0.00	4.13	5.37	3.40	4.68	4.49	4.79	0.00	3.31	4.77	5.32	3.22	2.33
G	2.70	4.35	0.00	2.61	4.63	0.00	0.00	3.56	2.29	3.75	4.31	0.00	4.08	3.64	3.61	2.24	0.00	4.34	4.38	0.00	0.00
24D_F2 2 mm																					
R	97.66	94.63	94.70	84.79	75.48	89.86	93.14	89.61	87.33	90.22	85.05	91.07	90.42	91.06	90.71	92.81	93.85	89.83	88.77	92.28	97.07
T	0.65	0.00	2.06	4.32	6.33	4.27	2.75	0.00	3.34	0.00	3.48	3.18	2.56	0.00	3.44	2.03	2.73	0.00	3.02	2.86	1.17
A	0.72	1.07	1.18	3.96	0.00	1.18	1.45	2.57	3.70	1.81	0.00	1.39	0.00	1.56	0.00	1.82	1.05	1.86	0.00	1.25	0.87
C	0.00	1.78	2.07	0.00	8.16	4.69	2.66	2.60	0.00	2.83	5.93	4.36	3.24	2.53	2.99	0.00	2.37	3.16	4.32	3.61	0.89
G	0.97	2.52	0.00	6.93	10.03	0.00	0.00	5.23	5.63	5.13	5.53	0.00	3.77	4.85	2.85	3.35	0.00	5.15	3.90	0.00	0.00
24D_R2 2mm																					
R	97.62	95.41	93.89	95.86	90.00	91.16	91.31	92.60	94.20	90.03	88.28	87.85	88.29	89.24	86.82	94.41	86.51	84.90	82.04	83.58	96.21
T	0.70	0.00	2.24	0.96	2.53	3.01	2.91	0.00	1.48	0.00	2.50	3.65	3.01	0.00	3.87	1.30	4.33	0.00	3.81	5.43	1.41
A	0.70	0.94	1.60	1.37	0.00	2.01	2.13	1.83	1.77	2.12	0.00	2.77	0.00	2.42	0.00	2.10	3.03	3.41	0.00	4.07	1.04
C	0.00	1.76	2.27	0.00	4.57	3.83	3.65	3.30	0.00	4.08	6.05	5.72	5.77	5.13	6.23	0.00	6.13	6.66	9.87	6.92	1.34
G	0.98	1.89	0.00	1.82	2.91	0.00	0.00	2.27	2.54	3.77	3.17	0.00	2.92	3.21	3.08	2.19	0.00	5.03	4.28	0.00	0.00
